# Major transcriptomic differences are induced by warmer temperature conditions experienced during asexual and sexual reproduction in *Fragaria vesca* ecotypes

**DOI:** 10.3389/fpls.2023.1213311

**Published:** 2023-07-14

**Authors:** Yupeng Zhang, Marcos Viejo, Igor Yakovlev, Torstein Tengs, Paal Krokene, Timo Hytönen, Paul E. Grini, Carl Gunnar Fossdal

**Affiliations:** ^1^Department of Molecular Plant Biology, Norwegian Institute of Bioeconomy Research, Ås, Norway; ^2^EVOGENE, Department of Biosciences, University of Oslo, Oslo, Norway; ^3^Department of Functional Biology, University of Santiago de Compostela, Santiago de Compostela, Spain; ^4^Department of Agricultural Sciences, Viikki Plant Science Centre, University of Helsinki, Helsinki, Finland

**Keywords:** *Fragaria vesca*, asexual (clonal) reproduction, sexual reproduction, epigenetics, transcriptome (RNA-seq)

## Abstract

A major challenge for plants in a rapidly changing climate is to adapt to rising temperatures. Some plants adapt to temperature conditions by generating an epigenetic memory that can be transmitted both meiotically and mitotically. Such epigenetic memories may increase phenotypic variation to global warming and provide time for adaptation to occur through classical genetic selection. The goal of this study was to understand how warmer temperature conditions experienced during sexual and asexual reproduction affect the transcriptomes of different strawberry (*Fragaria vesca*) ecotypes. We let four European *F. vesca* ecotypes reproduce at two contrasting temperatures (18 and 28°C), either asexually through stolon formation for several generations, or sexually by seeds (achenes). We then analyzed the transcriptome of unfolding leaves, with emphasis on differential expression of genes belonging to the epigenetic machinery. For asexually reproduced plants we found a general transcriptomic response to temperature conditions but for sexually reproduced plants we found less significant responses. We predicted several splicing isoforms for important genes (e.g. a SOC1, LHY, and SVP homolog), and found significantly more differentially presented splicing event variants following asexual vs. sexual reproduction. This difference could be due to the stochastic character of recombination during meiosis or to differential creation or erasure of epigenetic marks during embryogenesis and seed development. Strikingly, very few differentially expressed genes were shared between ecotypes, perhaps because ecotypes differ greatly both genetically and epigenetically. Genes related to the epigenetic machinery were predominantly upregulated at 28°C during asexual reproduction but downregulated after sexual reproduction, indicating that temperature-induced change affects the epigenetic machinery differently during the two types of reproduction.

## 1 Introduction

Plant adaptation to changing abiotic conditions is crucial for seasonal performance, reproductive success and, ultimately, species survival. Many plants are already facing rapidly increasing temperatures due to global warming and one adaptation strategy plants can use is to increase their plasticity, i.e. the ability to rapidly adjust the phenotype to fit environmental conditions (Nicotra et al., 2010). According to quantitative genetics, phenotypic variation results only from the genotype, environmental variation, and their interaction. However, we now know that epigenetic changes also contribute to phenotypic variation and this calls for a reexamination of the underlying nature of environmentally caused variance in plant traits (Hirsch et al., 2012; Diez et al., 2014).

When it comes to plant phenology, the best documented case of a truly long lasting epigenetic memory induced by warmer temperatures is found in the long-lived gymnosperm *Picea abies* (reviewed in Kvaalen and Johnsen, 2008; Yakovlev et al., 2012; Bräutigam et al., 2013). In addition to adaptation through classical Mendelian selection, *P. abies* adjusts its phenology to local temperature conditions depending on the temperature sum experienced during zygotic embryogenesis (sexual reproduction) (Kvaalen and Johnsen, 2008; Yakovlev et al., 2012; Bräutigam et al., 2013). This temperature memory leads to e.g. altered bud phenology and frost hardiness in the resulting epitypes (i.e., genetically identical plants that differ epigenetically) (Johnsen et al., 2005; Skrøppa et al., 2007). The same epigenetic temperature memory is also observed when spruce plants are propagated asexually through somatic embryogenesis (Kvaalen and Johnsen, 2008). At the transcriptomic level, this epigenetic memory is reflected in differential expression of mRNAs and non-coding RNAs that impact numerous genes, including genes in the epigenetic machinery, siRNA pathway, and phenology-regulating genes such as *FT-like 2* (*FTL2*), *PaDCL1 and 2, PaSGS3* and *EARLY BUD BREAK 1* (*EBB1*) (Yakovlev et al., 2010; Yakovlev et al., 2011; Yakovlev et al., 2014; Carneros et al., 2017). Thus, in the Norway spruce example, significantly warmer conditions during the time period for embryogenesis and seed maturation are likely a key factor driving rapid adaptation through epigenetic mechanisms.

The most studied epigenetic mark in plants is DNA methylation, i.e. 5’-methylation of cytosine. DNA methylation is known to provide plasticity to important phenotypes, such as drought and salt tolerance and in *Arabidopsis thaliana* (Arabidopsis) (Zhang et al., 2013; Kooke et al., 2015). This plasticity might be mediated by DECREASE IN DNA METHYLATION I (DDM1) (Zhang et al., 2013; Kooke et al., 2015). Furthermore, when Arabidopsis is treated with the demethylation agent 5-azacytidine several phenotypes are affected, including flowering time and growth rate in some genotypes (Bossdorf et al., 2010). Treating early-flowering lines of woodland strawberry (*Fragaria vesca*) Hawaii-4 plants with 5-azacytidine leads to up- or downregulation of several transcripts (Xu et al., 2016a; Xu et al., 2016b). The downstream genes of the flowering pathway *FLOWERING LOCUS T* (*FT*), *NUCLEAR FACTOR Y SUBUNIT B 2* (*NFYB2*), *FRUITFULL* (*FUL*), and *SEPALLATA 3* (*SEP3*) are upregulated, while *CONSTANS* (*CO*) and *CYCLING DOF FACTORs* (*CDFs*), *CIRCADIAN 1* (*CIR1*), *LATE ELONGATED HYPOCOTYL* (*LHY*), *CIRCADIAN CLOCK ASSOCIATED 1* (*CCA1*), and *PSEUDO-RESPONSE REGULATOR 9* (*PRR9*) related to photoperiod and the circadian clock are downregulated (Xu, 2016). Also, a recent study on three European *F. vesca* ecotypes suggests that DNA methylation is involved in local adaptation of this species (Sammarco et al., 2022).

*F. vesca* (2n = 2x = 14) occurs throughout Europe, Northern Asia, North America, and Northern Africa and is a perennial angiosperm that reproduces both sexually and asexually (through stolons). This reproductive flexibility may be important for the climatic adaptability of *F. vesca*, which is found across a wide range of latitudes and altitudes with very different temperature conditions. In a study including accessions from diverse habitats across the northern hemisphere, diploid species in Genus Fragaria including *F. vesca* displayed considerable differences in important phenological traits such as timing of flowering and stolon formation (Fan et al., 2022). Moreover, recently reported latitudinal clines in the timing of summer leaf senescence and winter leaf formation are other examples of adaptive phenological variation in *F. vesca* (Still et al., 2023). *F*. *vesca* is an attractive model species because it is relatively easy to propagate and has a relatively small genome that is already sequenced, making it amenable for molecular studies (Edger et al., 2018). The species is also of economical interest as a model species for the Rosaceae family and as a distant ancestor of the commercial strawberry *Fragaria × ananassa* (Shulaev et al., 2008; Liston et al., 2014).

­In this study we examined transcriptomic changes associated with an epigenetic memory of the temperature conditions experienced during asexual and sexual propagation in *F. vesca*. We analyzed transcriptomes of unfolding summer leaves of four European *F. vesca* ecotypes following sexual or asexual propagation at 18 or 28°C. We characterized the epigenetic temperature memory by identifying (1) differentially expressed genes (DEGs) using the latest *F. vesca* gene models, (2) DEGs in specific pathways and functional categories such as the epigenetic machinery, and (3) predicted alternative splicing events that may be influenced by temperature.

## 2 Materials and methods

### 2.1 Plant materials and experimental conditions

Four *Fragaria vesca* ecotypes (genotypes) [‘ES12’ (43.5339 °N, 6.5271 °W, 138 m, Spain), ‘ICE2’ (63.9988 °N, 19.9604 °W, 99 m, Iceland), ‘IT4’ (46.2398 °N, 11.2360 °E, 949 m, Italy) and ‘NOR2’ (69.9395 °N, 23.0964 °E, 23 m, Norway)] were grown in a growth chamber equipped with Valoya AP67 LED lamps [(200 μmol m^−2^s^−1^ photosynthetically active radiation (PAR)] at 40-45% humidity. A long day (LD) length of 16h/8h (light/dark) was used for plant vegetative growth, and a short day (SD) length of 12h/12h was used to induce flowering. Plants were grown in 400 ml pots and fertilized regularly using a fertilizer solution (N-P-K: 17-4-25, Kekkilä, Finland) (Zhang, 2022; Zhang et al., 2023).

**Figure 1:**
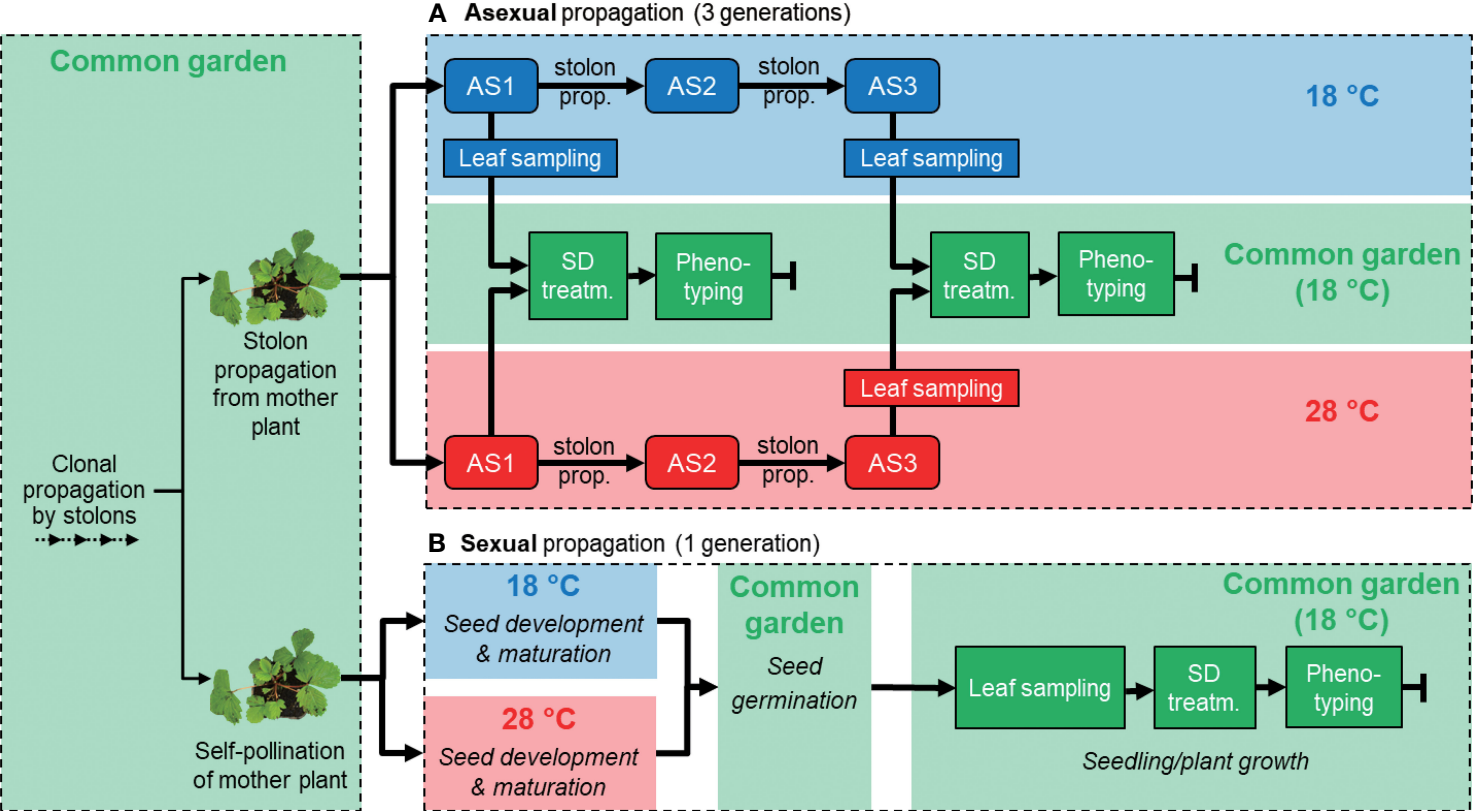
Experimental design: propagation, sample collection, and phenotypic observations of asexually **(A)** and sexually **(B)** propagated *Fragaria vesca* plants. **(A)** Four *F*. *vesca* ecotypes were propagated for three asexual generations (AS1-3) through stolon propagation. Plants were propagated at 18°C (blue boxes) and 28°C (red boxes). Phenotypic observations were made in a common garden environment (18°C; green boxes) after 5-6 weeks of short day treatment (SD). Leaf samples for RNA-seq were collected from AS1 and AS3. **(B)** The same four *F*. *vesca* ecotypes were propagated for one sexual generation through achenes. As in **(A)**, plants were propagated at 18 and 28°C and phenotypic observations were made in a common garden environment (18°C). Flowering time was observed after 6 weeks SD treatment. Leaf samples for RNA-seq were collected in the common garden.

Three asexual generations were propagated at 18 or 28 °C to induce epigenetic changes. The first asexual generation was obtained using stolons from mother plants of the four ecotypes and is referred to as asexual generation 1 (AS1). We used 10 plants for each combination of ecotype and temperature (n = 10). Subsequent asexual generations (AS2 and AS3) were obtained by using stolons of the previous asexual generation. Four weeks after the initiation of AS1 and AS3, 20 mg of young unfolded leaf was collected for RNA sequencing from three randomly selected plants per ecotype, 6 hours after the light period started (**Figure 1A**). All 10 plants per treatment combination were then transferred to a growth room to record phenotypic characters under common-garden conditions (LD, 18 °C). Flowering time was observed as days to the first open flower after the plants had been subjected to temporary SD treatment for 5 (AS1) or 6 weeks (AS3) to induce flowering. The rate of stolon formation on each plant was recorded every two weeks after SD treatment. Petiole length was measured one week after SD treatment as a proxy for plant growth.

For sexual reproduction, plants were placed individually in mesh bags made of a soft fabric (20 cm × 20 cm) that were closed at their base. The mesh allowed airflow but had a pore size that prevented movement of pollen between plants. Flowers were manually self-pollinated at 18 °C and LD conditions. Twenty-four hours later, plants were transferred to growth rooms at 18 or 28 °C to induce epigenetic changes from the early embryogenesis stage and onwards (Hollender et al., 2012). Mature achenes were later collected from berries and germinated on half MS media at 21 °C (Murashige and Skoog, 1962). Four weeks after germination, seedlings were potted and placed under common-garden conditions in a growth room (LD, 18 °C). For these sexually propagated plants we used 20 plants (grown from seeds) for each combination of ecotype and temperature (n = 20). After another four weeks, 20 mg of young unfolded leaf was collected for RNA sequencing from three randomly selected plants per ecotype, 6 hours after the light period started (**Figure 1B**). We recorded flowering time to assay phenology change, measured petiole length as a proxy for plant growth and recorded rate of stolon formation for all 20 plants per treatment combination in the same manner as for asexually propagated plants. In addition, we recorded the number of growth points during SD treatment.

#### 2.1.1 Isolation and sequencing of RNA

Extraction of total RNA from leaves of asexually reproduced plants was done as described previously (Zeng et al., 2018). RNA isolation from sexually reproduced plants was done using the same protocol (Zeng et al., 2018) until the first step of chloroform extraction. The remainder of the isolation was performed using the Spectrum™ Plant Total RNA Kit (Merck Ltd) following the manufacturer’s instructions. Transcriptome libraries were made and RNA-seq was performed on cDNA by BGI using and following the BGI’s DNBSEQ™ platform protocol. The generated reads are paired-end and length is 100bp.

#### 2.1.2 Statistical and bioinformatic analyses

**Figure 2:**
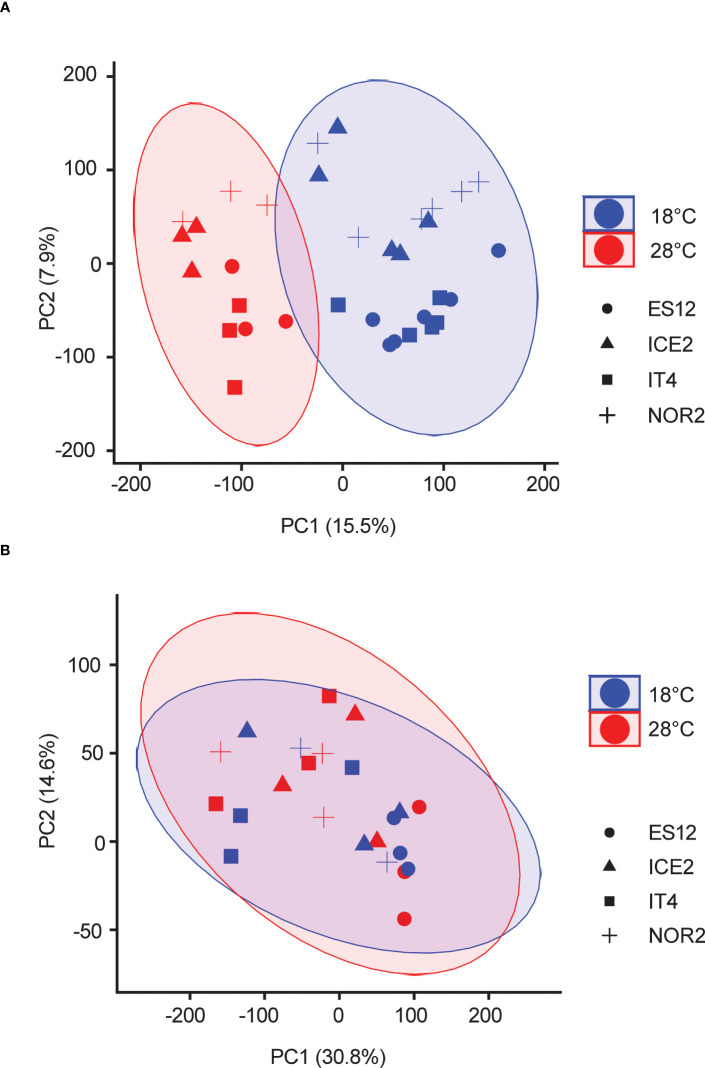
Principal component analysis of transcriptomes of four *Fragaria vesca* ecotypes (ES12, ICE2, NOR2, IT4) propagated asexually **(A)** or sexually **(B)** at 18 and 28 °C. Different symbols indicate ecotypes, different colors indicate temperature. Transcriptome data are from n = 3 biological replicates from each combination of ecotype and temperature treatment. Plants were propagated asexually for three generations and sexually for one generation.

The statistical analysis of flowering time, petiole length, and runner number was done by using Wilcoxon test in R. The read quality control was done by using fastqc (Andrews et al., 2010). RNA-seq data were analyzed using default settings in CLC Genomics Workbench (Qiagen Ltd). The paired reads were mapped to the latest *F.vesca* genome V4 (Edger et al., 2018). We used standard CLC Workbench setup so the analysis included 1-2% non-unique reads. These were randomly distributed. CLC Genomics Workbench uses trimmed mean of M values (TMM) for library size normalization and log counts per million (CPM) for RNA-seq normalization. Z-score normalization was used as cross-sample normalization and a generalized linear model was used to call differentially expressed genes (DEGs, |log2FoldChange| > 1.5, p-value < 0.05). Wald test was used for statistical testing of DEGs. Volcano plots of DEGs were generated using the ggplot2 package in R, based on log2(FoldChange values) and log10 (p-values) from the CLC output. Gene ontology (GO) term enrichment analysis was done using the R package clusterprofiler (Wu et al., 2021) and GO term annotation (v4.0.a2) (Li et al., 2019) was acquired from the Genome Database Rosaceae [GDR, https://www.rosaceae.org/, (Jung et al., 2004)]. The adjusted p-value (Benjamini-Hochberg Procedure, BH) of 0.01 was used as cutoff for GO term enrichment analysis. Epigenetics-related genes were identified by utilizing the GDR database and comparing the blastp output to known gene models from Arabidopsis and other model plant species (Jung et al., 2004). A GO term network analysis was performed using a ClueGO add-on in Cytoscape (Shannon et al., 2003; Bindea et al., 2009; Smoot et al., 2011). Predictions of alternative splicing events were made using the software rMATS (Shen et al., 2014). The STAR (2.7.10b) was used as a reads aligner for rMATS due to its sensitivity of detecting splicing events (Dobin et al., 2013). STAR ignores the reads that are mapped to more than 10 loci/locations. For reads mapped to 10 loci/location or less, STAR randomly distributes the reads. rMATS retrieves AS events from our obtained gene models and annotates them as AS types (e.g. SE, IR, A3SS) (Shen et al., 2014). It detects the possible AS events based on the genome annotation and calculates ψ, the exon inclusion level. ψ represents the proportion of reads supporting one AS outcome compared to the others (Shen et al., 2014). The range of ψ is always between 0 and 1 (Shen et al., 2014). rMATS then calculates the posterior probability of a significant difference, i.e. differential splicing events (DPAS), in exon inclusion level between the two temperature conditions using a user-defined cutoff, p(|ψ 1−ψ 2|>c | d a t a) (Shen et al., 2014). The adjusted p-value for DPAS is 0.01. PCA of individual replicates (for whole transcriptomes and DEGs) was done using prcomp() in R and the transcript per million (TPM) value of each gene (**Figures 2**, **S2**, **S8**).

#### 2.1.3 Accession numbers and supplemental data

All sequences generated in this study have been deposited in the National Center for Biotechnology Information Sequence Read Archive (https://www.ncbi.nlm.nih.gov/sra) under project numbers PRJNA879428 and PRJNA882853. Supplemental Data and Tables are available at GitHub (https://github.com/sherlock0088/FvTranscriptomes).

## 3 Results

Under identical (common garden) conditions we detected lasting phenotype changes in *F. vesca* plants that had experienced the warmer temperature earlier during their asexual and sexual reproduction (**Figure 1**). The ecotypes we studied were from Norway (NOR2), Iceland (ICE2), Spain (ES12), and Italy (IT4). Only NOR2 showed significant differences in flowering time between treatments following both asexual and sexual reproduction (**Figure S1**; **Table S1**). ICE2 showed significant temperature memory differences in flowering time following the treatment experienced only during asexual reproduction (**Table S1**). In ES12 and IT4 the rate of stolon formation differed significantly between temperature treatments, but only following asexual reproduction. ES12, as well as NOR2, differed significantly in the number of growth points following sexual reproduction at the warmer condition (**Figure S1**; **Table S1**).

### 3.1 Principal component analysis of transcriptomic data indicate both lasting temperature and ecotype-specific effects

RNA-seq data from unfolded leaves of *F. vesca* plants exposed to 18 or 28 °C during asexual and sexual reproduction were analyzed by PCA (**Figure 2**; **Table S2**). The transcriptomes of asexually reproduced plants separated according to temperature treatment. The largest principal component (PC1) separated plants reproduced at 18 or 28 °C and explained 15.5% of the variance (**Figure 2A**). PC2, which partly separated ecotypes (**Figure S2A**), explained only 7.9% of the variance (**Figure 2A**). For sexually propagated plants, PC1 and PC2 explained 30.8% and 14.6% of the variance, respectively. There were no clear temperature or ecotype effects for sexually propagated plants, as all biological replicates grouped together (**Figures 2B**, **S2B**). Taken together, PCA analyses of the entire transcriptome of sexually and asexually propagated *F. vesca* plants revealed major transcriptome reprogramming between temperature treatments only for asexually propagated plants. Ecotype-specific effects were less clear, but NOR2, ES12 and IT4 showed the more distinct grouping in asexually reproduced plants and ES12 in the sexually reproduced plants.

### 3.2 Warmer conditions experienced earlier during reproduction cause lasting differential gene expression in the resulting plants

**Figure 3:**
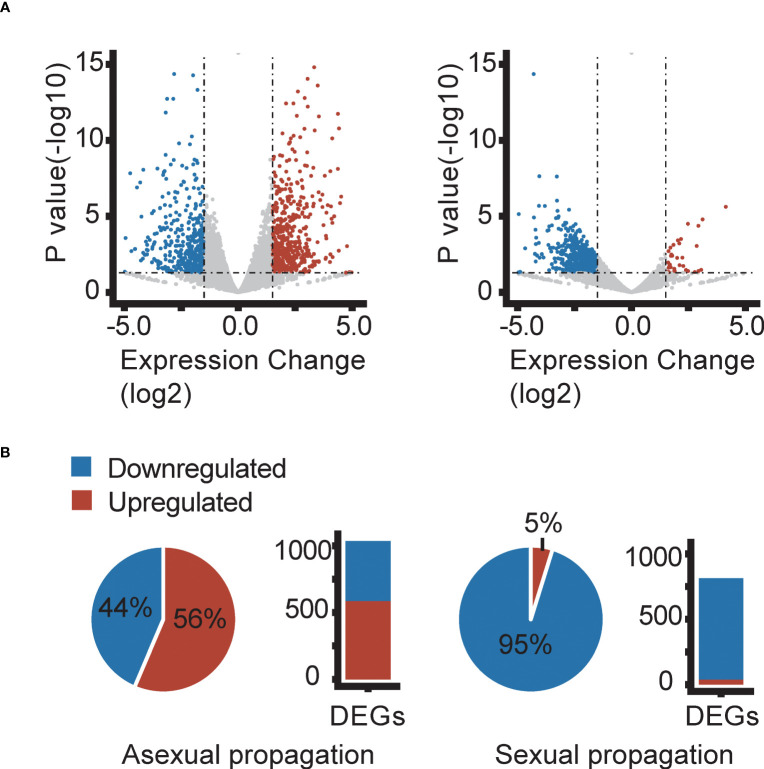
Differentially expressed genes (DEGs) in the *Fragaria vesca* NOR2 ecotype following asexual (left) and sexual (right) propagation at 18 or 28 °C. Plants were propagated asexually for three generations and sexually for one generation. Differential expression was calculated as gene expression in plants propagated at 28 °C relative to that in plants propagated at 18 °C. DEGs were called using P-value ≤ 0.05 and log_2_FoldChange ≥ |1.5| (horizontal and vertical dashed lines in volcano plots). **(A)** Volcano plots showing downregulated (blue) and upregulated (red) DEGs. **(B)** Proportions (pie charts) and absolute numbers (bar charts) of down- and upregulated DEGs. Transcriptome data are from n = 3 biological replicates per temperature treatment.

**Figure 4:**
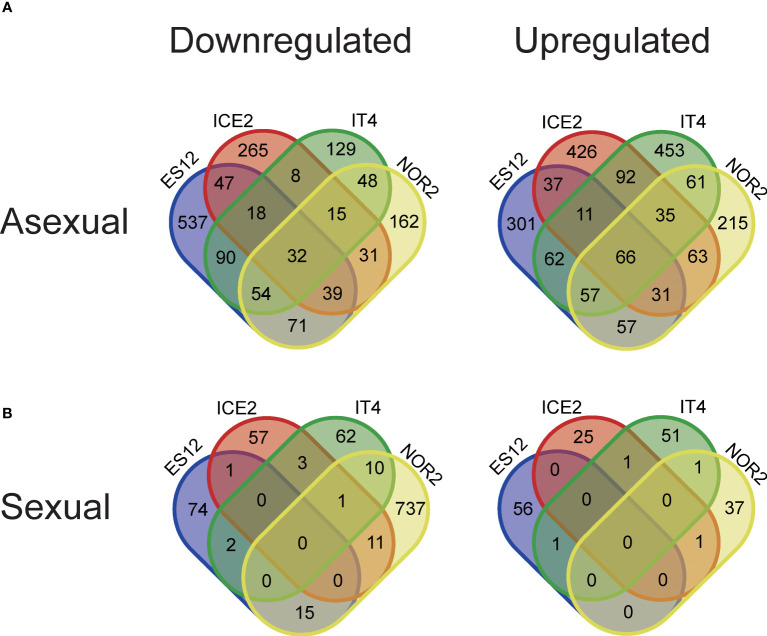
Venn diagram showing differentially expressed genes (DEGs) between four *Fragaria vesca* ecotypes (ES12, ICE2, NOR2, IT4) following asexual **(A)** and sexual **(B)** propagation at 18 and 28 °C. Differential expression was calculated as gene expression in plants propagated at 28 °C relative to that in plants propagated at 18 °C. Plants were propagated asexually for three generations and sexually for one generation. Venn diagrams show downregulated (left) and upregulated (right) DEGs in the different ecotypes. Transcriptome data are from n = 3 biological replicates from each combination of ecotype and propagation mode.

To identify genes involved in the temperature conditions experienced during sexual and asexual reproduction altered gene expression, we identified significantly differentially expressed genes (DEGs) between plants reproduced at 18 or 28 °C. In the third asexual generation (AS3), we found a total of 1037, 1510, 1216, and 1231 DEGs responding to elevated temperature (28 vs. 18 °C) in the ecotypes NOR2, ES12, ICE2, and IT4, respectively (**Figures 3**, **4**, **S3**–**S5**). Of these DEGs, 452, 888, 455, and 394 were downregulated and 585, 622, 761, and 837 were upregulated (**Figures 3**, **4**, **S3**–**S5**). In total, 32 downregulated and 66 upregulated DEGs were shared between all ecotypes (**Figure 4A**). We also compared the transcriptome of AS3 plants propagated at 28°C with that of AS1 and AS3 plants propagated at 18°C. In addition, AS1 plants propagated at 18°C was compared with that of AS3 plants propagated at 18°C to control and compare for any generational effects at this normal temperature, and found it to be significantly smaller than the impact of the warmer 28°C propagation condition. Thus, this cross-generational and between-temperature comparison confirmed the large effect elevated temperatures had on gene expression in the *F. vesca* ecotypes we studied with the exception of ICE2 (**Figures S4**–**S6**). The PCA of DEGs indicates that there are some ecotype specific effects with ES12 and IT4 being better separated from the other ecotypes while NOR2 and ICE2 show more overlap (**Figure S8A**).

Sexually propagated plants had much fewer significant DEGs between plants propagated at 18 or 28 °C (**Figure 3**). We found a total of 813, 149, 100, and 132 DEGs in NOR2, ES12, ICE2, and IT4, respectively (**Figures S3**–**S5**). Among these, 774, 92, 73, and 78 were downregulated and only 39, 57, 27, and 54 were upregulated (**Figures 3**, **4**, **S3**–**S5**). Not a single DEG was shared between all ecotypes in sexually propagated plants (**Figure 4B**). The NOR2 ecotype stood out, as it had five to eight times more DEGs than the other ecotypes and almost all of these (95.2%) were downregulated (**Figure 4**). The PCA of DEGs supports that there are ecotype specific effects also in the sexual experiment, particularly ES12 and NOR2 are very well separated from other ecotypes (**Figure S8B**).

### 3.3 Higher temperatures in asexually and sexually reproduced plants result in altered transcript levels in the epigenetic machinery

Given its role in the formation of an epigenetic temperature memory, we mined the transcriptomes of the four ecotypes for DEGs related to the epigenetic machinery. In asexually propagated plants we found 37 DEGs that potentially are involved in the establishment and maintenance of epigenetic marks under elevated temperatures. Of these, eight were downregulated and 29 were upregulated. Downregulated genes were related to chromatin remodeling (*SWIB/MDM2* domain in ES12), regulation of RNA-dependent DNA methylation (RdDM) pathways (*SAWADEE* domain in ES12, ICE2, and IT4), DNA methylation (*DEMETERr-like* gene in NOR2), and histone modification (*PHD-like* genes in ES12 and NOR2). Upregulated genes also included genes related to RdDM (*XH/XS* domain in all four ecotypes) and histone modification (*PHD-like* genes in all four ecotypes, *FRIGIDA-like* genes in ES12 and ICE2, and histone deacetylase-like genes in NOR2) (**Table S3**).

In sexually propagated plants we found fewer DEGs related to the epigenetic machinery than in asexually propagated plants (**Table S3**). This mirrored the pattern we found for the total number of DEGs. Only eight DEGs related to the epigenetic machinery were found, and all of these were downregulated. In ES12 we identified one DEG chromatin remodeling gene (chromatin remodeling 31), in IT4 we found a DNA demethylation gene (DNA glycosylase), and in NOR2 we found two histone H2A variants (i.e., H2A.Z; H2A9, H2A11), one histone methylation reader (tudor domain), three histone modification readers (PHD-like genes), one histone methyltransferase gene (SET domain), and one chromatin remodeling complex gene (SNF). No DEGs related to the epigenetic machinery were found in ICE2 (**Table S3**).

### 3.4 GO term analysis of DEGs in asexually reproduced plants show molecular function enrichment related to treatment and ecotype

**Figure 5:**
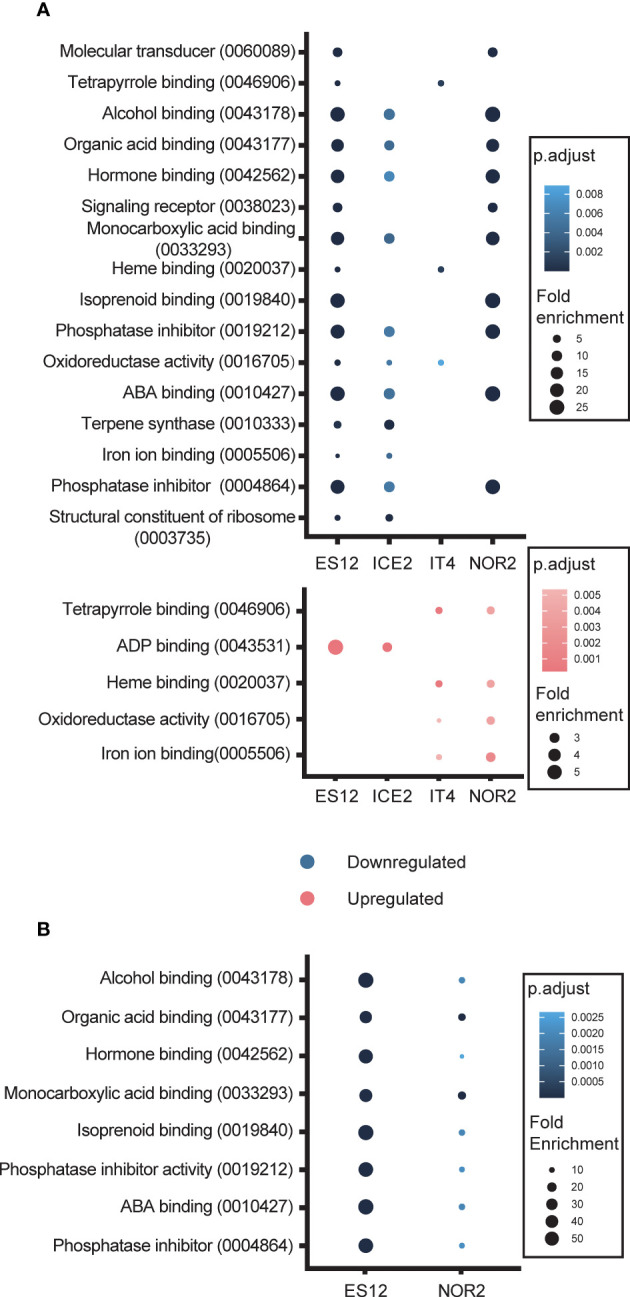
Gene Ontology (GO) terms enriched with differentially expressed genes in four *Fragaria vesca* ecotypes (ES12, ICE2, NOR2, IT4) following asexual **(A)** and sexual **(B)** propagation at 18 and 28 °C. Differential expression was calculated as gene expression in plants propagated at 28 °C relative to that in plants propagated at 18 °C. Plants were propagated asexually for three generations and sexually for one generation. GO terms, along with their digital identifier, are shown on the y-axes. Down- and upregulated GO terms are shown in blue and red, respectively. Color shading indicates adjusted p-value levels and dot size indicates fold-enrichment at 28 vs. 18 °C.

All four ecotypes (ES12, ICE2, IT4 and NOR2) displayed massive alteration in their transcriptome when they were propagated at elevated temperatures (28 vs. 18 °C). To better understand the transcripts underpinning this response, we first did a gene ontology (GO) analysis and found that enriched DEGs in the elevated temperature treatment fell within the ‘molecular function’ category of GO terms. Among downregulated DEGs, seven GO terms were shared by ES12, ICE2, and NOR2: alcohol binding, organic acid binding, hormone binding, monocarboxylic acid binding, phosphatase inhibitor, abscisic acid (ABA) binding, and phosphatase inhibitor (**Figure 5**; **Table S4**). IT4 had a different GO term-enrichment than the other ecotypes and shared only one enriched GO term with ES12 and ICE2 (oxidoreductase activity) and two with ES12 (tetrapyrrole binding and heme binding). IT4 also had a lower enrichment of GO terms than in all the other ecotypes.

Some of the enriched GO terms we detected were related to hormone responses to temperature stress, in particular ABA binding (**Figure 5A**). Also, among downregulated DEGs, 14 GO terms were enriched in only one ecotype (**Figure S9**): ES12 had seven unique GO terms, ICE2 had three, IT4 had one, and NOR2 had three. Of these 14 GO terms, chitinase activity (in ES12) and oxidoreductase activity (in NOR2) had the highest enrichment-fold level (**Table S4**). Both of these are involved in stress and defence responses.

Among upregulated DEGs, five GO terms were involved in ADP binding, iron ion binding, heme binding, tetrapyrrole binding, and oxidoreductase activity (reduction of molecular oxygen) (**Figure 5**; **Table S4**). All these GO terms were shared by two ecotypes. Interestingly, the GO term oxidoreductase activity included genes that were both up- and downregulated in IT4. Eight GO terms were enriched in only one ecotype (**Figures 5**, **S9**; **Table S4**).

We also checked for DEGs that were unique to each ecotype. Several unique downregulated genes were found in ES12. Here we found a *AGAMOUS-LIKE 8* (*AGL8*) homolog, a *SVP/JOINTLESS LIKE/AGL22* homolog, a *AP2/B3-like transcriptional factor* (*AP2/B3*) protein homolog, seven *WRKY protein homologs* (in the GO term GO:0003700 DNA-binding transcription factor activity), and six *S locus-related/S receptor homologs* (in the GO term GO:0004674 protein serine/threonine kinase activity). These *WRKY* and *S locus related/S receptor* homologs were also found in ICE2, but here they were upregulated, not downregulated (**Table S4**). The NOR2 ecotype was enriched in two oxidoreductase activities: one using diphenols and related substances as donors and oxygen as acceptor (GO:0016682), and the other using paired donors, where oxidation of the donors reduces molecular oxygen to two molecules of water (GO:0016717). These oxidoreductase activities are likely upregulated to remove reactive oxygen species (ROS) produced under stressful conditions (**Table S4**). Other GO terms that were enriched in NOR2 are related to lipid binding (GO:0008289) and antioxidant activity (GO:0016209) (**Table S4**).

### 3.5 GO term analysis of DEGs in sexually reproduced plants show enrichment of molecular functions related to treatment and ecotype

The four ecotypes exhibited altered transcript levels also when plants were propagated sexually at elevated temperatures, but unlike asexually propagated plants we did not find any GO terms that were enriched for upregulated transcripts (**Figure 5A**; **Table S4**). Eight GO terms enriched for downregulated transcripts were shared between NOR2 and ES12, with ES12 showing the highest enrichment-fold levels (**Figure 5B**; **Table S4**). These eight GO terms included ABA binding, isoprenoid binding, alcohol binding, protein phosphatase inhibitor, phosphatase inhibitor activity, hormone binding, monocarboxylic acid binding, and organic acid binding (**Figure 5B**; **Table S4**). Except for isoprenoid binding, all of these were also downregulated during asexual reproduction in ES12, NOR2, and ICE2 (**Figure 5A**; **Table S4**).

Only ES12 and NOR2 displayed ecotype-specific, downregulated GO terms in sexually propagated plants. These belonged to four GO terms: molecular transducer, signaling receptor, protein disulfide oxidoreductase, and calcium ion binding (**Figure S9B**; **Table S4**). These GO terms were enriched only in sexually propagated plants and not during asexual propagation.

### 3.6 GO categories related to ABA binding are enriched in asexually reproduced plants

**Figure 6:**
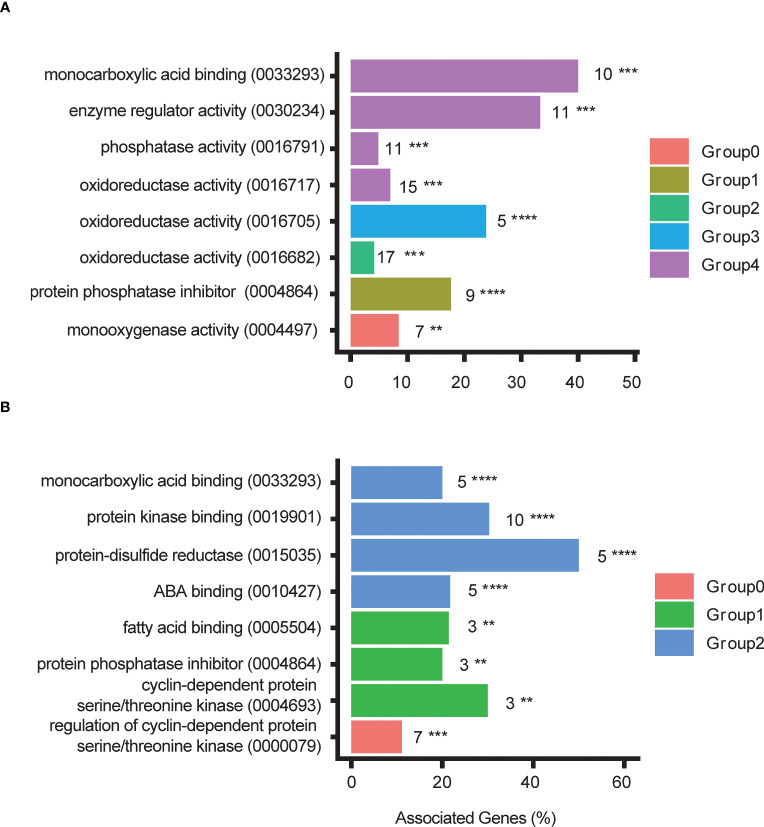
Gene Ontology (GO) terms and GO term groups enriched for differentially downregulated genes in the NOR2 *Fragaria vesca* ecotype following asexual **(A)** and sexual **(B)** propagation. Differential expression was calculated as gene expression in plants propagated at 28 °C relative to that in plants propagated at 18 °C. Plants were propagated asexually for three generations and sexually for one generation. GO terms and their digital identifier are shown on the y-axes. For each GO term the number of differentially expressed genes is given next to the bar. Colors indicate GO term groups. The x-axes indicate the proportion of genes inside each GO term that is differentially expressed Asterisks indicate significance: ** 0.001 ≤ p < 0.01; *** 0.0001 ≤ p < 0.001; **** 0.00001 ≤ p < 0.0001. ABA, abscisic acid.

To learn more about the categories involving DEGs in asexually propagated plants we used ClueGO on Cytoscape to group the representative GO terms and visualize them. Overall, different categories were enriched in different ecotypes (**Figures 6**, **S10**–**S12**; **Table S3**). For upregulated DEGs, the NOR2, ES12, ICE2, and IT4 ecotypes were enriched for three, three, six, and five different categories, respectively (**Figures 6**, **S10**–**S12**; **Table S3**). However, some enriched categories were shared by two or more ecotypes, such as oxidoreductase activity (acting on single donors and incorporating molecular oxygen) which was shared by ES12, IT4, and NOR2. Categories involving ADP-binding were enriched both in ES12 and ICE2, and the terpene synthesis category was enriched in IT4 and NOR2 (**Figures 6**, **S10**–**S12**; **Table S3**).

Most enriched categories involved downregulated DEGs. Downregulated DEGs in the NOR2, ES12, ICE2, and IT4 ecotypes were enriched in eight, three, six, and five different categories, respectively (**Figures 6**, **S10**–**S12**; **Table S3**). All ecotypes were enriched in the monocarboxylic acid-binding category (which includes the GO term abscisic acid-binding). ES12, IT4, and NOR2 also shared two enriched categories: terpene synthesis and oxidoreductase activity (acting on paired donors, incorporating or reducing molecular oxygen) (**Figures 6**, **S10**–**S12**; **Table S3**).

### 3.7 GO categories related to ABA binding are enriched in sexually propagated plants in response to the warmer conditions experienced earlier during reproduction

We also identified categories enriched with DEGs in sexually propagated plants. However, due to the low number of DEGs in ICE2 and IT4, these ecotypes were not enriched for any categories. The same was true for upregulated DEGs in NOR2.

For upregulated DEGs in ES12, only the terpene synthase category was enriched (**Figures 6**, **S10**–**S12**; **Table S5**). For downregulated DEGs, two categories were enriched in ES12: the terpene synthesis category and the protein phosphatase inhibitor category (**Figure S10**). Notably, transcripts in the protein phosphatase inhibitor category are involved in ABA-binding. For downregulated DEGs in NOR2, three categories were enriched: monocarboxylic acid-binding, protein kinase-binding, and protein-disulfide reductase activity (**Figure 6**; **Table S5**). Interestingly, the monocarboxylic acid-binding category includes both protein phosphatase inhibitors and ABA activity.

### 3.8 Alternative splicing events involved in response to warmer conditions experienced earlier during reproduction

Our *in silico* analysis of the *F. vesca* transcriptome revealed massive alternative splicing events. In the different ecotypes we detected 11,376 to 17,531 skipped exon (SE) sites, 895 to 2,150 mutually exclusive exon (MXE) sites, 24,119 to 33,260 retained intron (RI) sites, 26,397 to 40,533 alternative 3’ splicing sites (A3SS), and 15,321 to 22,785 alternative 5’ splicing sites (A5SS). The ES12 ecotype had the highest, and IT4 had the lowest, number of alternative splicing events. NOR2 had most A3SS events (40,533), A5SS events (22,785), RI events (33,260), and SE events (17,531), while ES12 had most MXE events (2,150) (**Figure S13**).

Overall, the number of alternative splicing events was lower in sexually propagated plants than in asexually propagated plants (8 to 48% lower for the different splicing events in each ecotype). To see how the temperature conditions experienced during reproduction influenced alternative splicing we compared the number of ecotype-specific differentially presented splicing events (DPAS) in plants propagated asexually and sexually at 28 or 18 °C. MXE was the most common differentially presented splicing event, making up 30% of the total number of DPAS. Among ecotypes, NOR2 had the largest difference in DPAS at 28 vs. 18 °C following both sexual and asexual reproduction. ES12 had the smallest difference.

**Figure 7:**
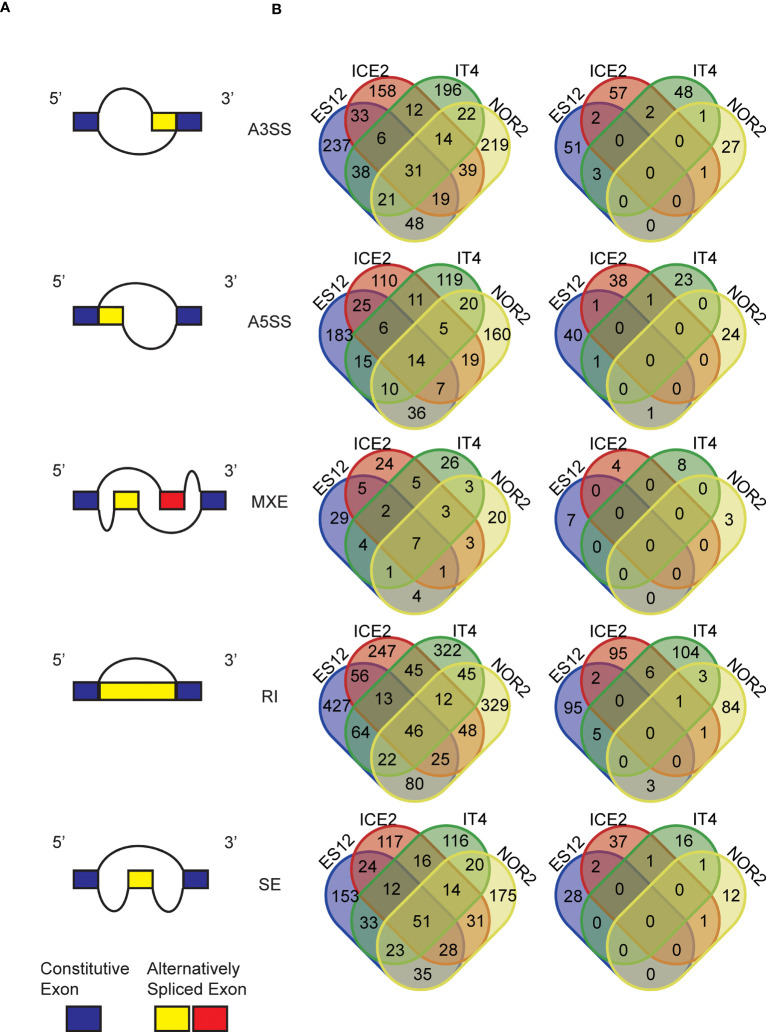
Alternative splicing events and differentially presented alternative splicing events (DPAS) in four *Fragaria vesca* ecotypes (ES12, ICE2, NOR2, IT4) following asexual and sexual propagation at 18 and 28 °C. **(A)** Different forms of alternative splicing events: A3SS = alternative 3’ splicing site; A5SS = alternative 5’ splicing site; MXE = mutually exclusive exon site; RI = retained intron site; SE = skipped exon site. **(B)** Venn diagrams show numbers of DPAS in plants propagated at 28 vs. 18 °C across ecotypes. Left: plants propagated asexually for three generations; right: plants propagated sexually for one generation.

The number of ecotype-specific DPAS between plants propagated at 28 vs. 18 °C was high in asexually propagated plants and low in sexually propagated plants. This mirrored the higher number of alternative splicing events overall in asexually propagated plants. Among all detected DPAS, we recorded 31 A3SS events, 14 A5SS events, seven MXE events, 46 RI events, and 51 ES events that were shared by all ecotypes in asexually propagated plants (**Figure 7**). In contrast, sexually propagated plants shared very few DPAS between several ecotypes, and not a single DPAS was shared among all ecotypes (**Figure 7**). In asexually propagated plants the percentage of DPAS detected at 28 vs. 18 °C tended to be below 5% for all splicing events in all ecotypes. The only exception was MXE, where the percentage varied from 3.8% up to 7.3% in the different ecotypes. However, in sexually reproduced plants, the percentage of most DPAS at 28 vs. 18 °C was well below 1% in all ecotypes, with MXE having the highest levels (0.31%-0.89%).

At the single gene level, the most common *in silico*-predicted DPAS were for *SUPPRESSOR OF OVEREXPRESSION OF CONSTANS 1* (*SOC1*), *LATE ELONGATED HYPOCOTYL* (*LHY*), and *SHORT VEGETATIVE PHAGE* (*SVP*). *SOC1* displayed mostly A3SS events, while *LHY* and *SVP* had mostly RI events (**Tables S6**-**16**).

## 4 Discussion

Global warming is a serious challenge for plants, as they must adapt to rapidly changing environmental conditions. Epigenetic change in the genome regulates gene expression and causes transcriptome reprogramming, thereby increasing phenotypic variation that natural selection may act upon. This epigenetic alteration creates genetically similar but phenotypically distinct individuals (epitypes) (Dwivedi et al., 2011; Feng and Jacobsen, 2011). Some plants generate epitypes in response to the temperature conditions experienced during sexual and asexual reproduction and these epitypes have an epigenetic memory of the environmental conditions experienced during embryogenesis (Kvaalen and Johnsen, 2008; Yakovlev et al., 2012; Bräutigam et al., 2013). Such a molecular memory is an epigenetic mechanism that may increase the phenotypic variation in response to climate change. Here, our results suggest that elevated temperature conditions during sexual and asexual reproduction cause lasting phenotypic change in combination with altered transcriptomes indicative of epigenetic memory effects in European *Fragaria vesca* ecotypes.

Transcriptome analysis of early-flowering *F. vesca* lines that had been hypomethylated following 5-azacytidine treatment indicates that flowering-related genes were either up- or downregulated, while genes related to the circadian clock were downregulated (Xu, 2016). From our own comparative transcriptome analysis we found that some epigenetic mechanisms and molecular function categories involved in adaptation to elevated temperature were affected when four different *F. vesca* ecotypes were propagated at elevated temperatures (28 vs. 18°C). Importantly, elevated temperatures caused more changes in the transcriptomic profile of asexually propagated plants than of sexually propagated plants. Among sexually propagated plants only one ecotype (NOR2) showed a distinct transcriptomic reprogramming. The reason for the greater transcriptomic response of asexually propagated plants might be due to longer exposure to elevated temperature for three consecutive generations, whereas sexually propagated plants were exposed for one generation (and only during embryogenesis and seed development). Transcriptomic responses of *F. vesca* to elevated temperatures during reproduction were also largely ecotype-specific. Within each ecotype, transcriptome changes (DEGs) between plants propagated at 28 vs. 18°C tended to be more pronounced than the DEG similarity to the other ecotypes.

Most of the genes that were impacted by elevated temperature conditions (28°C) during asexual reproduction are involved in hormone responses and terpene synthesis. Among the hormone-related genes, we found many abscisic acid (ABA)-related genes, including ABA synthesis and ABA receptor genes, downregulated in different ecotypes. ABA plays important roles in growth and stress responses in plants, specifically in the general response to high temperatures (43°C), including growth restriction in response to environmental challenges (Suzuki et al., 2016). Moreover, ABA may increase heat tolerance by improving the plants’ antioxidant capacity and buffering against increasing levels of reactive oxygen species (ROS) to prevent protein denaturation, misfolding and aggregation (Goraya et al., 2017; Lippmann et al., 2019). However, the thermotolerance regulatory network mediated by ABA is still poorly known in *F. vesca*. Besides ABA-related genes, genes related to oxidoreductase activity and terpene synthesis were also overrepresented among DEGs in plants propagated at elevated temperatures. We therefore believe that not only the plant hormone ABA, but also terpene synthesis may be affected in responses to elevated temperature in *F. vesca*. Terpenes, or terpenoids, make up the most chemically, structurally, and functionally varied metabolite group in living organisms. In plants, terpenes have important roles, for example as gibberellins and membrane sterols. Terpene synthesis transcripts are known to be influenced by high temperatures and hormones, as well as other complex compounds, are implicated in the thermotolerance regulatory network in plants (Zhou and Pichersky, 2020).

We found some DEGs related to the epigenetic machinery, suggesting that altered epigenetic marks are involved in *F. vesca’s* responses to elevated temperature. The fact that the four ecotypes we studied had distinct sets of epigenetics-related DEGs following reproduction at elevated temperatures suggests a strong degree of genetic fine-tuning in the induction of the epigenetic memory. Alternatively, there may be significant allelic differences between ecotypes in epigenetic machinery genes and their promoters. We found epigenetics-related DEGs involved in RdDM and DNA demethylation, histone modification and reading, and chromatin remodeling. DEGs also included histone variants like XH/XS-domain genes that can process double-stranded RNA to siRNA (an essential step in the RdDM pathway; (Finke et al., 2012), SAWADEE-domain genes that also can act as lysine readers by binding to unmethylated histone H3 lysine 4 and 9 (H3K4 and H3K9) in the RdDM pathway (Law et al., 2011; Law et al., 2013), and FRIGIDA-like genes that act as histone acetyltransferases in the COMPASS-like complex and can regulate the chromatin status near *FLOWERING LOCUS C* (*FLC*) region and further regulate *FLC*’s expression (Schmitz et al., 2005; Jiang et al., 2009). Furthermore, epigenetics-related DEGs included plant homeodomain-containing (PHD) genes that can act as histone modification readers recognizing lysine-methylated histone H3 (Sanchez and Zhou, 2011), SET domain genes involved in lysine methylation (Malagnac et al., 2002; Dillon et al., 2005), DEMETER like 1 and a DNA glycosylase involved in active DNA demethylation through base-excision-repair (BER) (Ikeda and Kinoshita, 2009), and SWIB/MDM2 and SNF domain genes belonging to the chromatin remodeler complex (the SWI/SNF complex). Also, some of the DEGs we found have been reported to have regulatory functions during temperature stress, such as the H2A variant H2A.Z. Histone variants can be recruited by the thermosensor PHYTOCHROME INTERACTING FACTOR 4 (PIF4) (Brickner et al., 2007; Xue et al., 2021).

Several studies show that alternative splicing can be an important stress-response mechanism in plants by integrating various environmental stimuli and diversifying the plant’s transcriptome and ultimately the proteome (Shang et al., 2017; Dikaya et al., 2021; Liu et al., 2022). Our *in silico* analysis of differentially presented alternative splicing events (DPAS) in *F. vesca* revealed that asexually propagated plants had significantly more DPAS than sexually propagated plants at elevated temperature. We found many splicing isoforms for some important genes, including *SOC1*, *LHY*, and a *SHORT VEGETATIVE PHASE* (*SVP*) homolog. Interestingly, these isoforms were among the most common *in silico*-predicted DPAS between temperatures. SOC1 is implicated in suppressing flowering and prolonging the vegetative growth period in *F. vesca* (Mouhu et al., 2013). It is thought to operate through the *TERMINAL FLOWERING LIKE 1* (TFL)-*APETALA1* (*AP1*) pathway to suppress flowering and through GA biosynthetic pathway to activate stolon formation, suggesting that SOC1 has a central role in photoperiodic control of both generative growth (i.e. sexual propagation through seeds) and vegetative growth (i.e asexual propagation through stolons) (Mouhu et al., 2013; Andrés et al., 2021; Fan et al., 2022). Alternative splicing may result in altered SOC1 interaction with other proteins modifying its regulatory role in inputs from photoperiods, gibberellin, and FLC or result in a less translatable transcript lowering SOC1 protein levels similarly to that reported by Song and co-workers (Song et al., 2009). Only one *LHY* homolog has been identified in the *F. vesca* genome, with < 50% amino acid sequence similarity to *AtLHY*. It is not yet known whether or not this putative *FvLHY* functions as a circadian clock gene regulating flowering and development rhythms in *F. vesca*. In Arabidopsis, several temperature-associated isoforms of LHY have been characterized. Splicing of the 5′UTR region of *LHY* is proposed to act as a molecular thermostat and the ratio of different transcript isoforms is temperature-dependent (James et al., 2018). Independent verification of isoforms and functional analysis of *LHY* isoforms in *F. vesca* is needed to ensure the actual function of *LHY* in response to changing temperatures.

FLOWERING LOCUS M (FLM) and SVP belong to the MADS-box proteins and regulate flowering in a combinatorial way in many plants. The *FLM* transcript is already known to be subjected to temperature-dependent alternative splicing in Arabidopsis (Shang et al., 2017; Liu et al., 2022). There are no previously reported splice isoforms of *SVP* in *F. vesca*, and we propose that the splicing variants we detected in different *F. vesca* ecotypes could affect dimer formation and other SVP/FLM protein interactions and thereby regulate flowering in a temperature-sensitive manner. Thus, the protein products resulting from alternative splicing variants could affect binding of the FLM-SVP complex to the promoter of the flowering time integrator *SOC1* (John et al., 2021) and thereby alter the time to flowering. We found five predicted *SVP* homologs in the *F. vesca* genome, one on chromosome Fvb1, two on Fvb4, and two on Fvb5. The homolog with the highest amino acid similarity to singular Arabidopsis *SVP* was on chromosome Fvb1 (> 88% similarity). The putatively least similar homolog was on Fvb5, with an amino acid similarity < 63%. The presence of multiple *SVP* homologs (and splicing variants thereof) in *F. vesca* indicates that these *SVP*s may have different roles from those present in Arabidopsis. Functional studies are needed to confirm the possible divergent role(s) and spatial and temporal expression patterns of different *F. vesca* homologs and their phenotypic impacts.

In conclusion, the results presented in this study point to a distinct transcriptomic response to the warmer temperature conditions experienced during reproduction in *F. vesca*. Since we are not exposing the plants to what is considered high temperature stress, we consider these responses to be an adaptation to warning conditions. The response was less distinct (statistically significant) in sexually propagated individuals, possibly because of the stochastic character of recombination during meiosis and/or due to differential creation or erasure of epigenetic marks during embryogenesis and seed development. Further deciphering of the genetic and epigenetic components of this response during meiosis and embryogenesis is needed. Our most striking finding was the low number of differentially expressed genes (DEGs) that were shared between ecotypes, suggesting that there is a strong genetic impact on the ability of different ecotypes to generate phenotypic variation. Moreover, the temperature conditions experienced during reproduction impact the epigenetic machinery in the *F. vesca* ecotypes. DEGs related to the epigenetic machinery were mostly upregulated by elevated temperatures during asexual propagation but downregulated during sexual propagation. Warmer temperature thus seems to impact the epigenetic machinery in a contrasting manner during the two modes of reproduction. Our *in silico* predictions showed significantly more differentially presented splicing event variants in response to elevated temperatures during asexual compared to sexual propagation. We predicted several potentially functionally divergent splicing isoforms for important genes, including *SOC1*, *LHY*, and *SVP*. Experimental confirmation and functional characterization of the possible roles of these key genes and their splice variants in epigenetic responses to warmer temperature in *F. vesca* will be a future priority.

## Data availability statement

The datasets presented in this study can be found in online repositories. The names of the repository/repositories and accession number(s) can be found in the article/**Supplementary Material**.

## Author contributions

CF designed the research; CF, PG, and YZ designed the analysis; YZ performed experiments; YZ, TT, IY, TH, PG, and CF analyzed the data; YZ, TT, TH, PG, and CF discussed the data; YZ, MV, PG, and CF wrote the article; YZ, MV, PK, TH, PG, and CF revised the article. All authors approved the article.

## Supplementary material

Supplementary Figure 1 Phenotypic differences between Fragaria vesca plants propagated asexually and sexually propagated from germinated seeds produced at epitype inducing conditions of 18 or 28 °C. Phenotypes were scored under common-garden conditions after AS1 and AS3 for the asexually propagated plants and plants from seed in the sexual experiment after flower-inducing short-day (SD) treatment. Dot size indicates -log_10_(p-value) value. Color indicates the increased or decreased values for 28°C condition. Panels along the x-axis are AS1 generation, AS3 generation in asexual reproduction, and Sexual reproduction. Asterisks indicate significances: * 0.01 ≤ p < 0.05; ** 0.001 ≤ p < 0.01; *** 0.0001 ≤ p < 0.001; **** 0.00001 ≤ p < 0.0001.

Supplementary Figure 2 Principal component analysis of transcriptomes of all three biological replicates in four *Fragaria vesca* ecotypes (ES12, ICE2, NOR2, IT4) propagated asexually **(A)** or sexually **(B)** at 18 and 28 °C. Different colors indicate ecotype and different symbols indicate temperature. Plants were propagated asexually for three generations and sexually for one generation.

Supplementary Figure 3 Differentially expressed genes (DEGs) in four *Fragaria vesca* ecotypes (ES12, ICE2, IT4, NOR2) propagated asexually at 18 and 28 °C. Differential expression was calculated as gene expression in plants propagated for three generations at 28 °C relative to that in plants propagated for one generation at 18 °C. Volcano plots show downregulated (blue) and upregulated (red) DEGs. DEGs were called using P-value ≤ 0.05 and log_2_FoldChange ≥ |1.5| (horizontal and vertical dashed lines). Pie charts show proportions of down- and upregulated DEGs. Transcriptome data are from n = 3 biological replicates from each combination of ecotype and propagation mode.

Supplementary Figure 4 Differentially expressed genes (DEGs) in three *Fragaria vesca* ecotypes (ES12, ICE2, IT4) propagated asexually or sexually at 18 and 28 °C. Differential expression was calculated as gene expression in plants propagated at 28 °C relative to that in plants propagated at 18 °C. Plants were propagated asexually for three generations and sexually for one generation. Volcano plots show downregulated (blue) and upregulated (red) DEGs. DEGs were called using P-value ≤ 0.05 and log_2_FoldChange ≥ |1.5| (horizontal and vertical dashed lines). Pie charts show proportions and bar charts show absolute numbers of down- and upregulated DEGs.

Supplementary Figure 5 Differentially expressed genes (DEGs) in four *Fragaria vesca* ecotypes (ES12, ICE2, NOR2, IT4) propagated asexually or sexually at 18 versus 28 °C. Asexually propagated plants were propagated for one or three generations (AS1 and AS3, respectively). Venn diagrams compare all DEGs, downregulated DEGs, and upregulated DEGs across ecotypes. All DEGs in AS1 vs. AS3 at both 18 and 28 °C are included.

Supplementary Figure 6 Differentially expressed genes (DEGs) in four *Fragaria vesca* ecotypes (ES12, ICE2, NOR2, IT4) propagated asexually for one (AS1) or three (AS3) generations at 18 and 28°C. Venn diagrams compare all DEGs, downregulated DEGs, and upregulated DEGs across ecotypes. Differential expression was calculated as gene expression in AS1 vs. AS3 plants propagated at 18 °C **(A)** and in AS3 plants propagated at 28 °C vs. AS1 plants propagated at 18 °C **(B)**.

Supplementary Figure 7 Differentially expressed genes (DEGs) in four *Fragaria vesca* ecotypes (ES12, ICE2, NOR2, IT4) propagated asexually for three generations (AS3) or sexually at 18 and 28°C. Differential expression was calculated as gene expression in plants propagated at 28 °C relative to that in plants propagated at 18 °C. Venn diagrams compare all DEGs across ecotypes following asexual propagation for three generations and sexual propagation for one generation.

Supplementary Figure 8 Principal component analysis of DEGs of all three biological replicates in four *Fragaria vesca* ecotypes (ES12, ICE2, NOR2, IT4) propagated asexually **(A)** or sexually **(B)** at 18 and 28 °C. Differential expression was calculated as gene expression in plants propagated at 28 °C relative to that in plants propagated at 18 °C. Plants were propagated asexually for three generations and sexually for one generation. Different colors indicate ecotype.

Supplementary Figure 9 Ecotype specific Gene Ontology (GO) terms enriched with differentially expressed genes in *Fragaria vesca* ecotypes (ES12, ICE2, NOR2, IT4) following asexual **(A)** and sexual propagation **(B)** at 18 and 28 °C. Differential expression was calculated as gene expression in plants propagated at 28 °C relative to that in plants propagated at 18 °C. GO terms, along with their digital identifier, are shown on the y-axes. Down- and upregulated GO terms are shown in blue and red, respectively. Color shading indicates adjusted p-value level and dot size indicates fold-enrichment at 28 vs. 18 °C.

Supplementary Figure 10 Gene Ontology (GO) terms and GO term groups enriched with differentially down- or upregulated genes in the ES12 *Fragaria vesca* ecotype following asexual and sexual propagation at 18 and 28 °C. Differential expression was calculated as gene expression in plants propagated at 28 °C relative to that in plants propagated at 18 °C. GO terms and their digital identifier are shown on the y-axes. For each GO term the number of differentially expressed genes is given next to the bar. Colors indicate GO term groups. The x-axes indicate the proportion of genes inside each GO term that is differentially expressed. Asterisks indicate significances: ** 0.001 ≤ p < 0.01; *** 0.0001 ≤ p < 0.001; **** 0.00001 ≤ p < 0.0001. FAD = flavin adenine dinucleotide; ADP = adenosine diphosphate; ATP = adenosine triphosphate.

Supplementary Figure 11 Gene Ontology (GO) terms and GO term groups enriched with differentially down- or upregulated genes in the ICE2 *Fragaria vesca* ecotype following asexual propagation at 18 and 28 °C. Differential expression was calculated as gene expression in plants propagated at 28 °C relative to that in plants propagated at 18 °C. GO terms and their digital identifier are shown on the y-axes. For each GO term the number of differentially expressed genes is given next to the bar. Colors indicate GO term groups. The x-axes indicate the proportion of genes inside each GO term that is differentially expressed. Asterisks indicate significance: ** 0.001 ≤ p < 0.01; *** 0.0001 ≤ p < 0.001; **** 0.00001 ≤ p < 0.0001. ATP = adenosine triphosphate; ADP = adenosine diphosphate.

Supplementary Figure 12 Gene Ontology (GO) terms and GO term groups enriched with differentially down- or upregulated genes in the IT4 *Fragaria vesca* ecotype following asexual propagation at 18 and 28 °C. Differential expression was calculated as gene expression in plants propagated at 28 °C relative to that in plants propagated at 18 °C. GO terms and their digital identifier are shown on the y-axes. For each GO term the number of differentially expressed genes is given next to the bar. Colors indicate GO term groups. The x-axes indicate the proportion of genes inside each GO term that is differentially expressed. Asterisks indicate significances: ** 0.001 ≤ p < 0.01; *** 0.0001 ≤ p < 0.001; **** 0.00001 ≤ p < 0.0001.

Supplementary Figure 13 Differentially presented alternative splicing events (DPAS) in four *Fragaria vesca* ecotypes (NOR2, IT4, ICE2, ES12) propagated asexually or sexually at 18 and 28 °C. Bars show numbers of DPAS for different forms of alternative splicing events in plants propagated at 28 vs. 18 °C. A3SS = alternative 3’ splicing sites; A5SS = alternative 5’ splicing sites; MXE = mutually exclusive exon sites; RI = retained intron sites; SE = skipped exon sites.
