# Supplementary material for: Major transcriptomic differences are induced by warmer temperature conditions experienced during asexual and sexual reproduction in Fragaria vesca ecotypes
Source: Front Plant Sci. 2023 Jul 14;14:1213311. doi: 10.3389/fpls.2023.1213311 (PMC10379642; doi:10.3389/fpls.2023.1213311)
Supplement: Supplementary Figure 1 — Phenotypic differences between Fragaria vesca plants propagated asexually and sexually propagated from germinated seeds produced at epitype inducing conditions of 18 or 28 °C. Phenotypes were scored under common-garden conditions after AS1 and AS3 for the asexually propagated plants and plants from seed in the sexual experiment after flower-inducing short-day (SD) treatment. Dot size indicates -log10(p-value) value. Color indicates the increased or decreased values for 28°C condition. Panels along the x-axis are AS1 generation, AS3 generation in asexual reproduction, and Sexual reproduction. Asterisks indicate significances: * 0.01 ≤ p < 0.05; ** 0.001 ≤ p < 0.01; *** 0.0001 ≤ p < 0.001; **** 0.00001 ≤ p < 0.0001. [file Image_1.pdf]

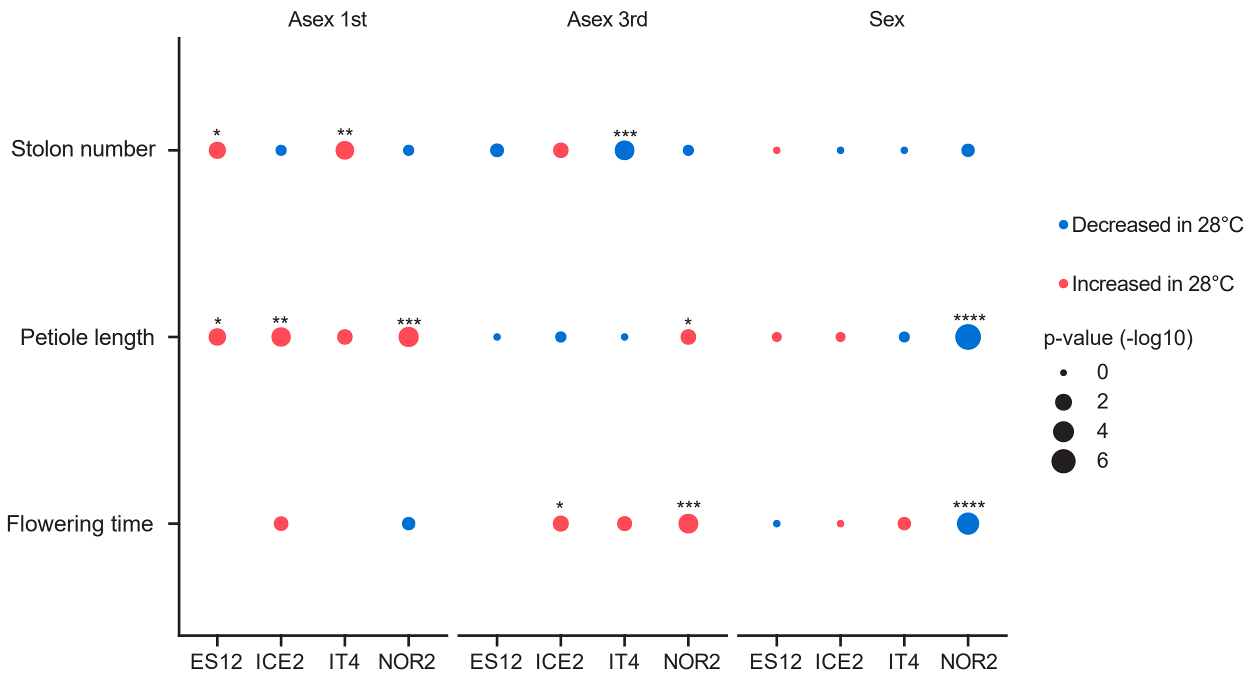
Asex 1st
Asex 3rd
Sex
Stolon number
Petiole length
Flowering time
ES12
ICE2
IT4
NOR2
ES12
ICE2
IT4
NOR2
ES12
ICE2
IT4
NOR2
Decreased in 28°C
Increased in 28°C
p-value (-log10)
0
2
4
6

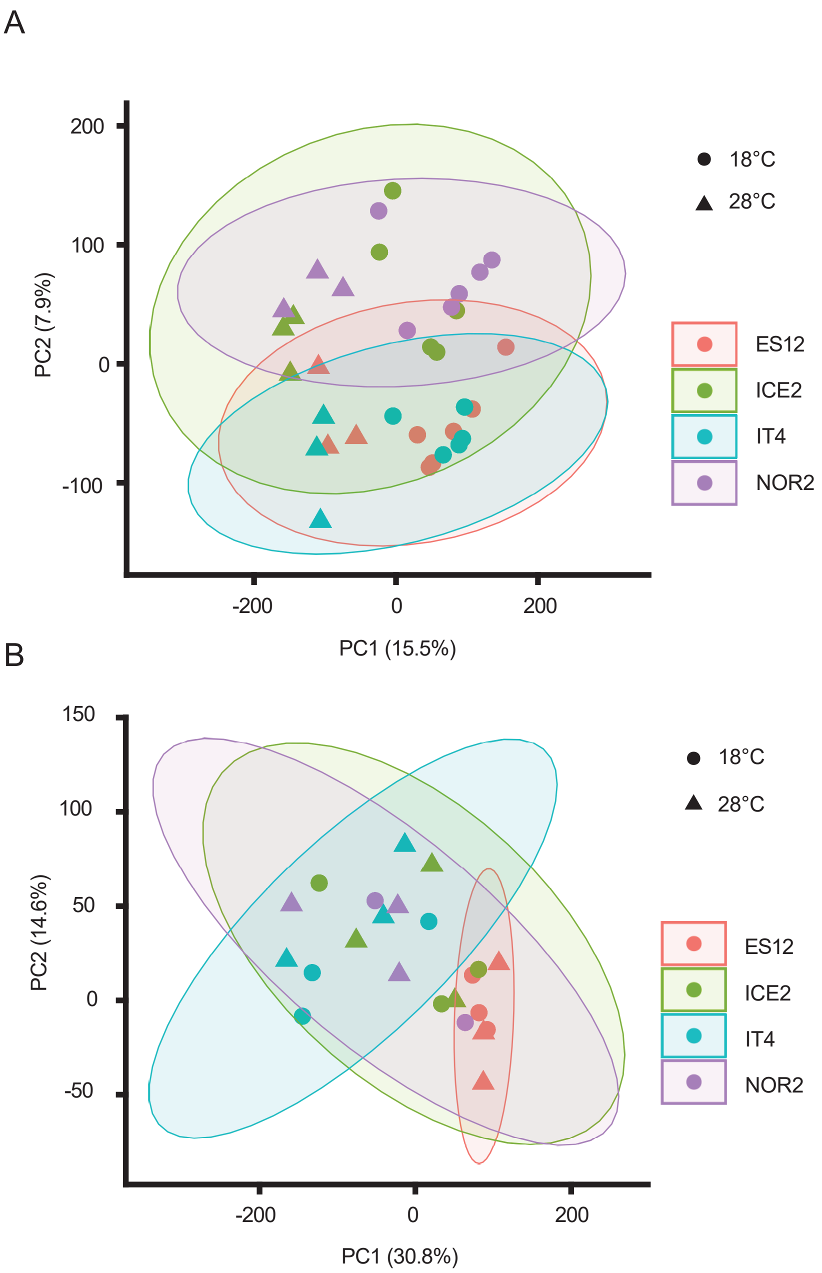
A
200
100
0
-100
-200
0
200
PC2 (7.9%)
PC1 (15.5%)
18°C
28°C
ES12
ICE2
IT4
NOR2
B
150
100
50
0
-50
-200
0
200
PC2 (14.6%)
PC1 (30.8%)
18°C
28°C
ES12
ICE2
IT4
NOR2

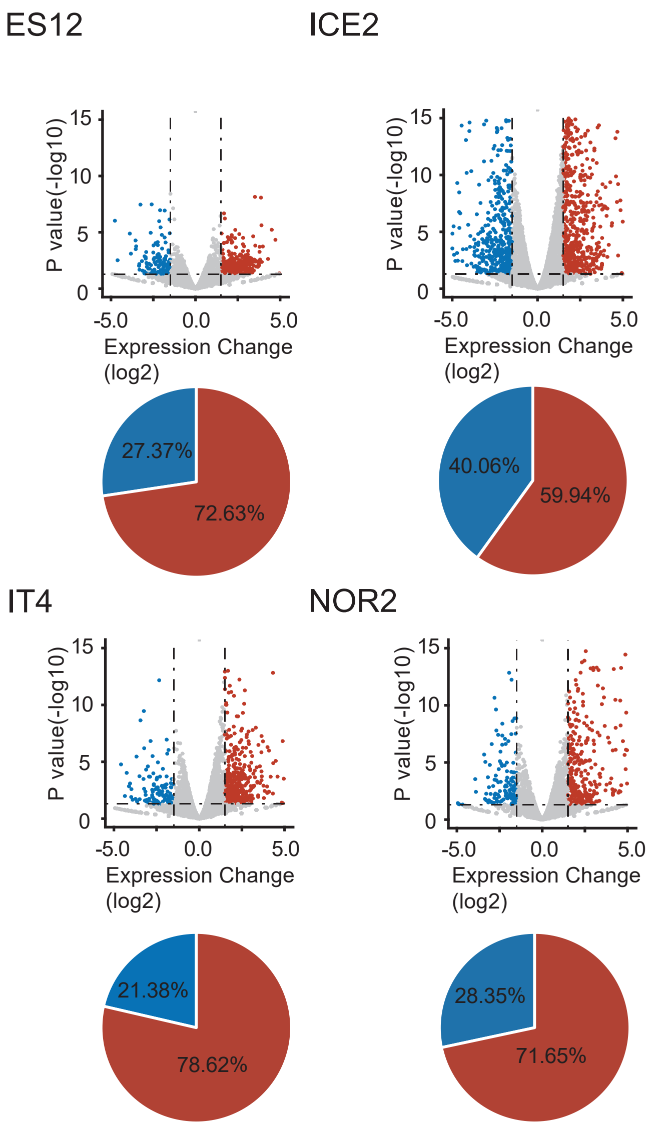
ES12
ICE2
P value(-log10)
15
10
5
0
-5.0
0.0
5.0
Expression Change
(log2)
27.37%
72.63%
40.06%
59.94%
IT4
NOR2
21.38%
78.62%
28.35%
71.65%

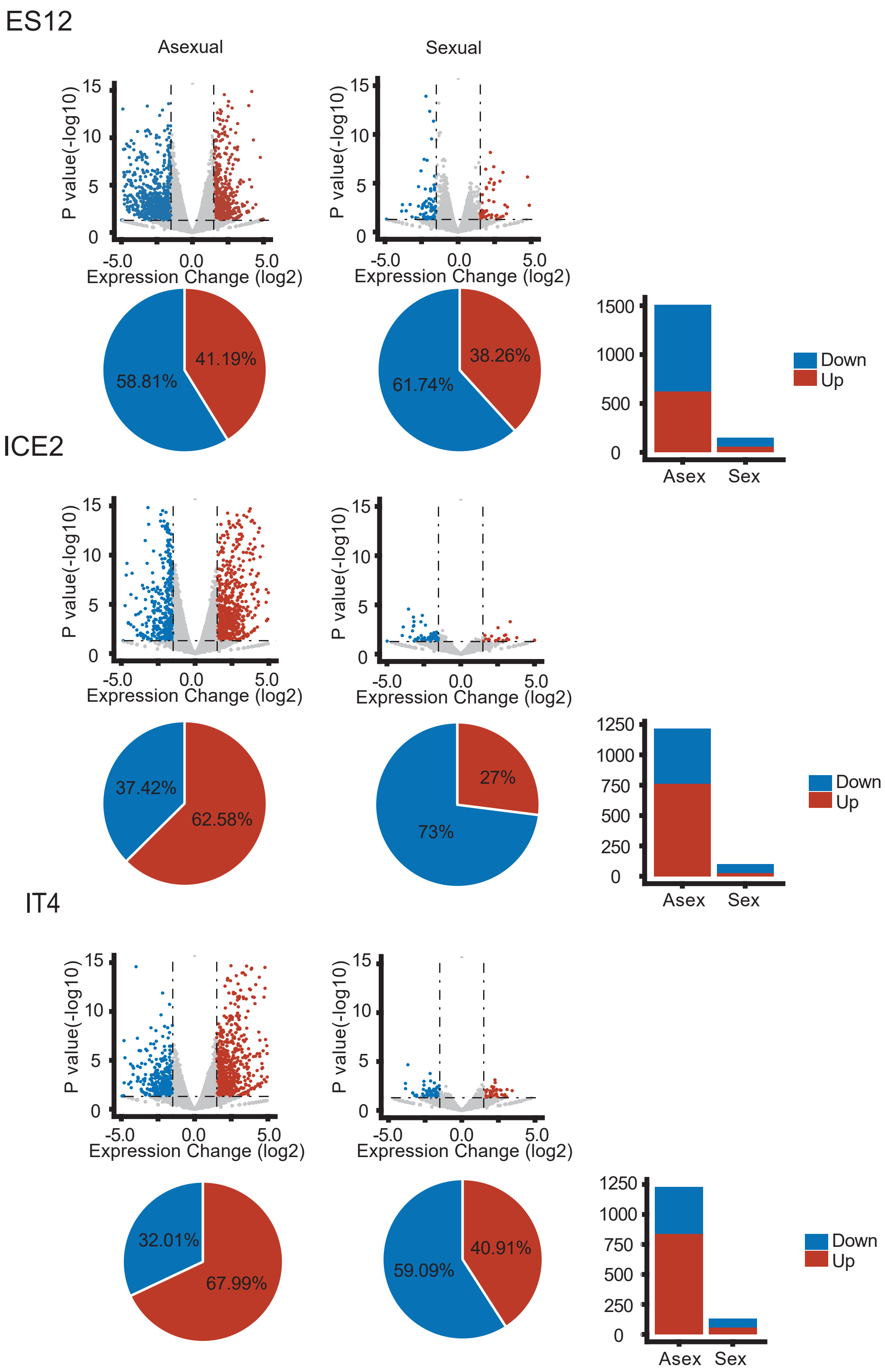
ES12
Asexual
Sexual
P value(-log10)
Expression Change (log2)
58.81%
41.19%
61.74%
38.26%
Asex
Sex
Down
Up
ICE2
37.42%
62.58%
27%
73%
IT4
32.01%
67.99%
59.09%
40.91%

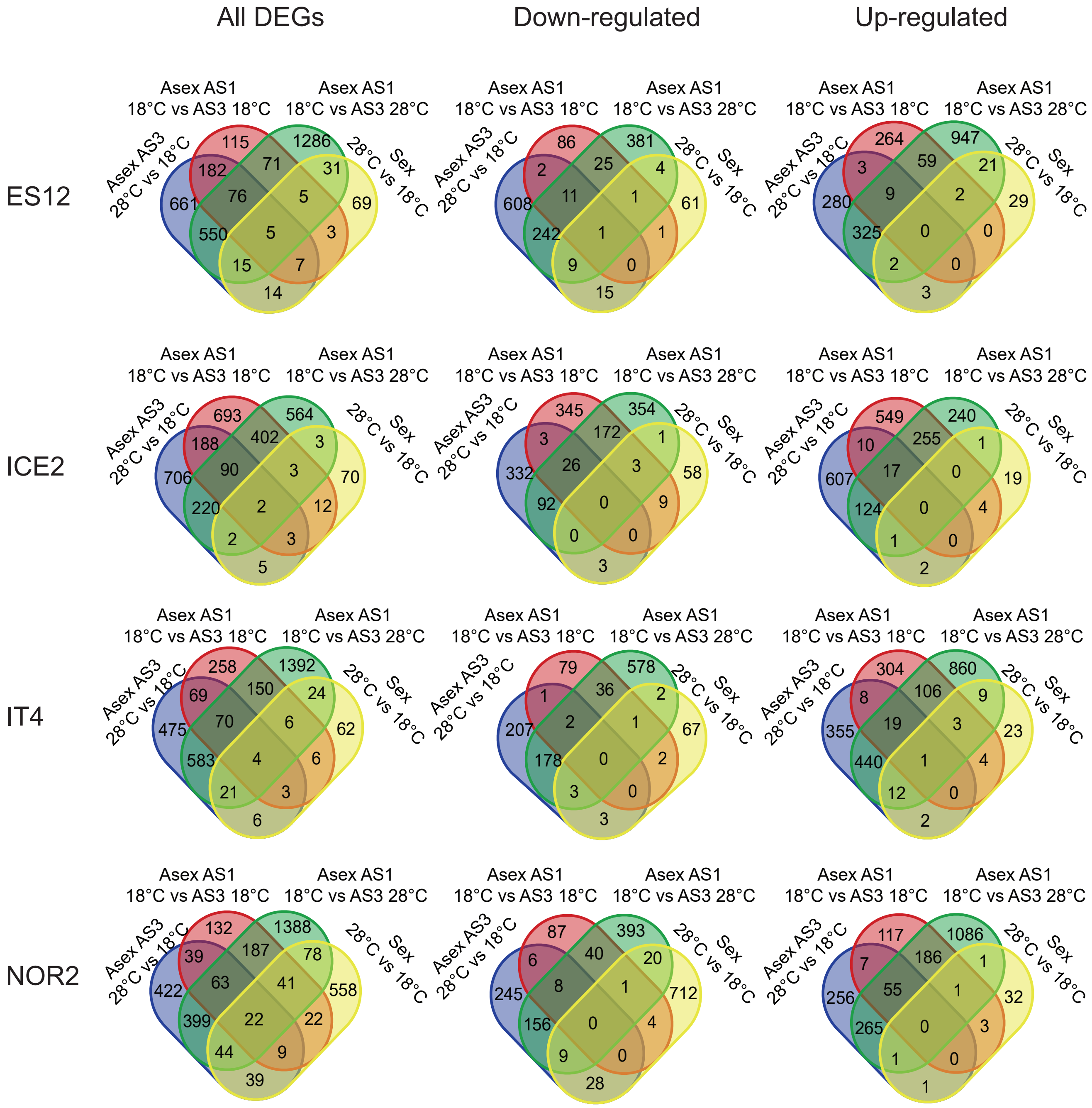

All DEGs
Down-regulated
Up-regulated
ES12
ICE2
IT4
NOR2
Asex AS1 18°C vs AS3 18°C
Asex AS1 18°C vs AS3 28°C
Asex AS3 28°C vs 18°C
Sex 28°C vs 18°C
115 1286 182 71 31 661 76 5 69 550 5 3 15 7 14
86 381 2 25 4 608 11 1 61 242 1 1 9 0 15
264 947 3 59 21 280 9 2 29 325 0 0 2 0 3
693 564 188 402 3 706 90 3 70 220 2 12 2 3 5
345 354 3 172 1 332 26 3 58 92 0 9 0 0 3
549 240 10 255 1 607 17 0 19 124 0 4 1 0 2
258 1392 69 150 24 475 70 6 62 583 4 6 21 3 6
79 578 1 36 2 207 2 1 67 178 0 2 3 0 3
304 860 8 106 9 355 19 3 23 440 1 4 12 0 2
132 1388 39 187 78 422 63 41 558 399 22 22 44 9 39
87 393 6 40 20 245 8 1 712 156 0 4 9 0 28
117 1086 7 186 1 256 55 1 32 265 0 3 1 0 1

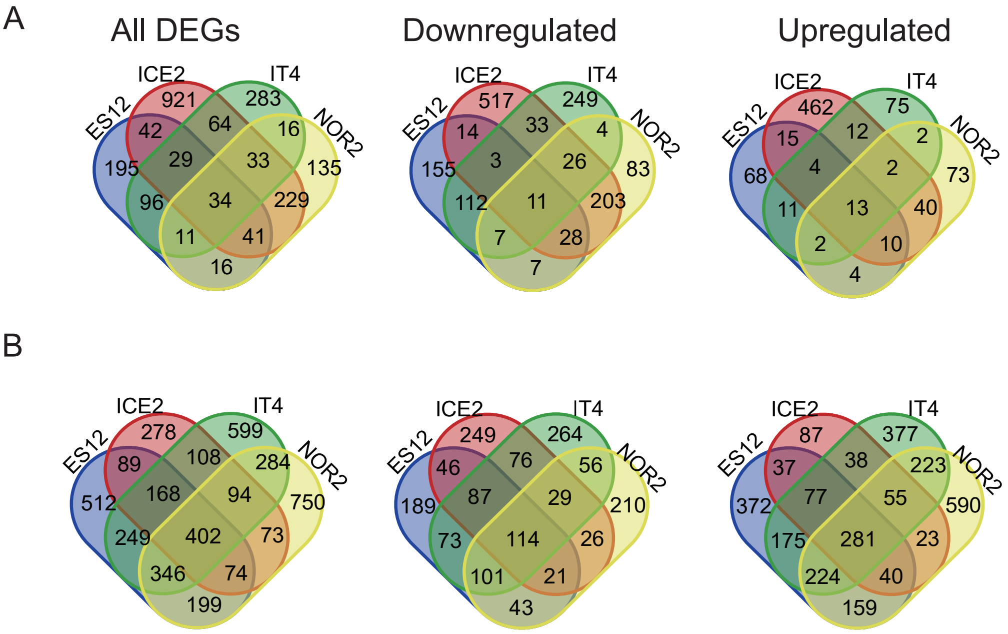
A
All DEGs
Downregulated
Upregulated
ICE2
IT4
ES12
NOR2
921
283
42
64
16
195
29
33
135
96
34
229
11
41
16
517
249
14
33
4
155
3
26
83
112
11
203
7
28
7
462
75
15
12
2
68
4
2
73
11
13
40
2
10
4
B
278
599
89
108
284
512
168
94
750
249
402
73
346
74
199
249
264
46
76
56
189
87
29
210
73
114
26
101
21
43
87
377
37
38
223
372
77
55
590
175
281
23
224
40
159

All DEGs

Asexual

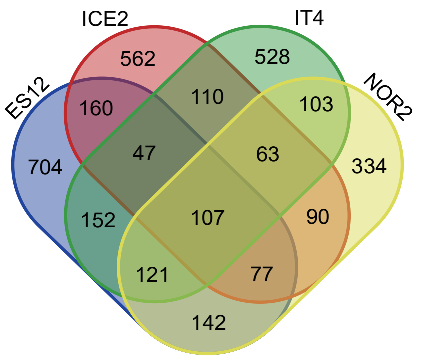

Sexual

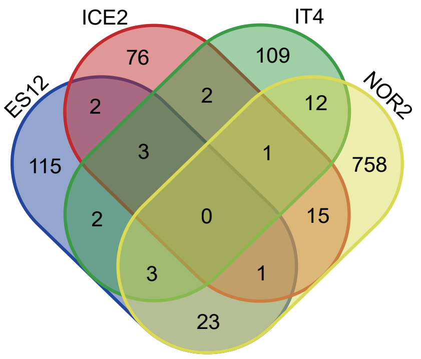

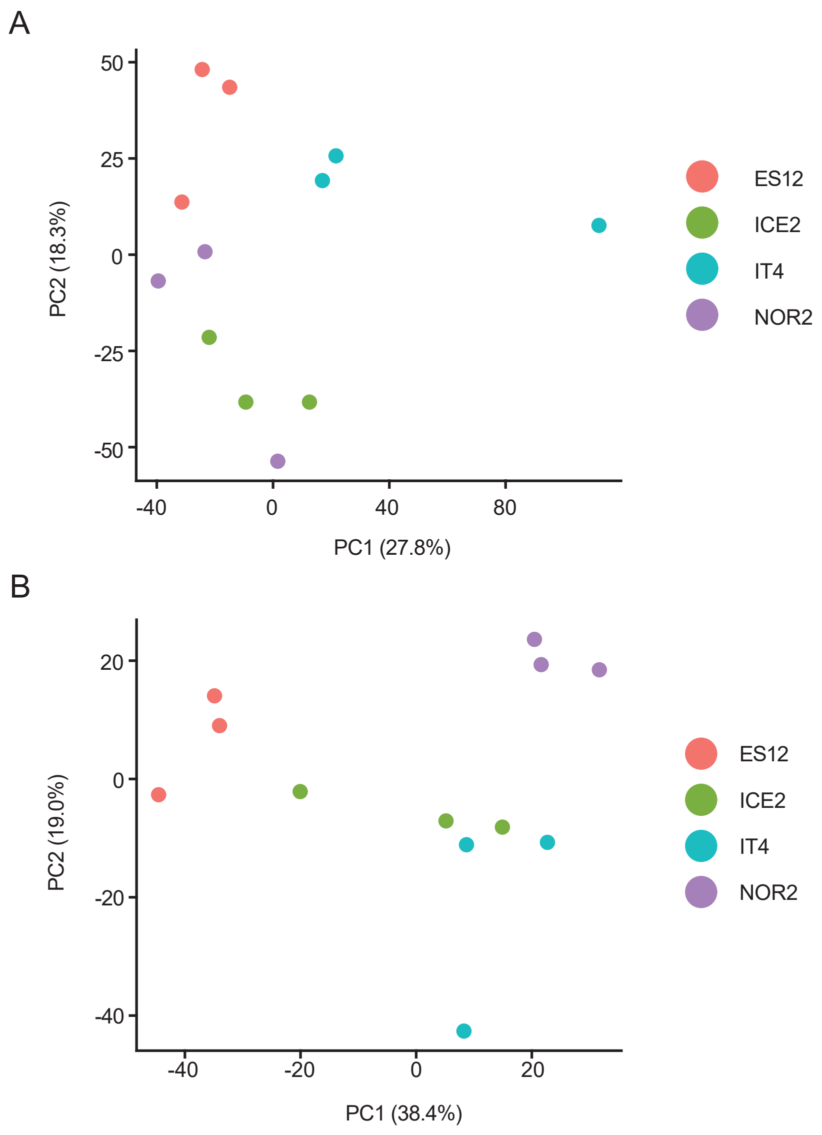
A
50
25
0
-25
-50
PC2 (18.3%)
-40
0
40
80
PC1 (27.8%)
ES12
ICE2
IT4
NOR2
B
20
0
-20
-40
PC2 (19.0%)
-40
-20
0
20
PC1 (38.4%)
ES12
ICE2
IT4
NOR2

A

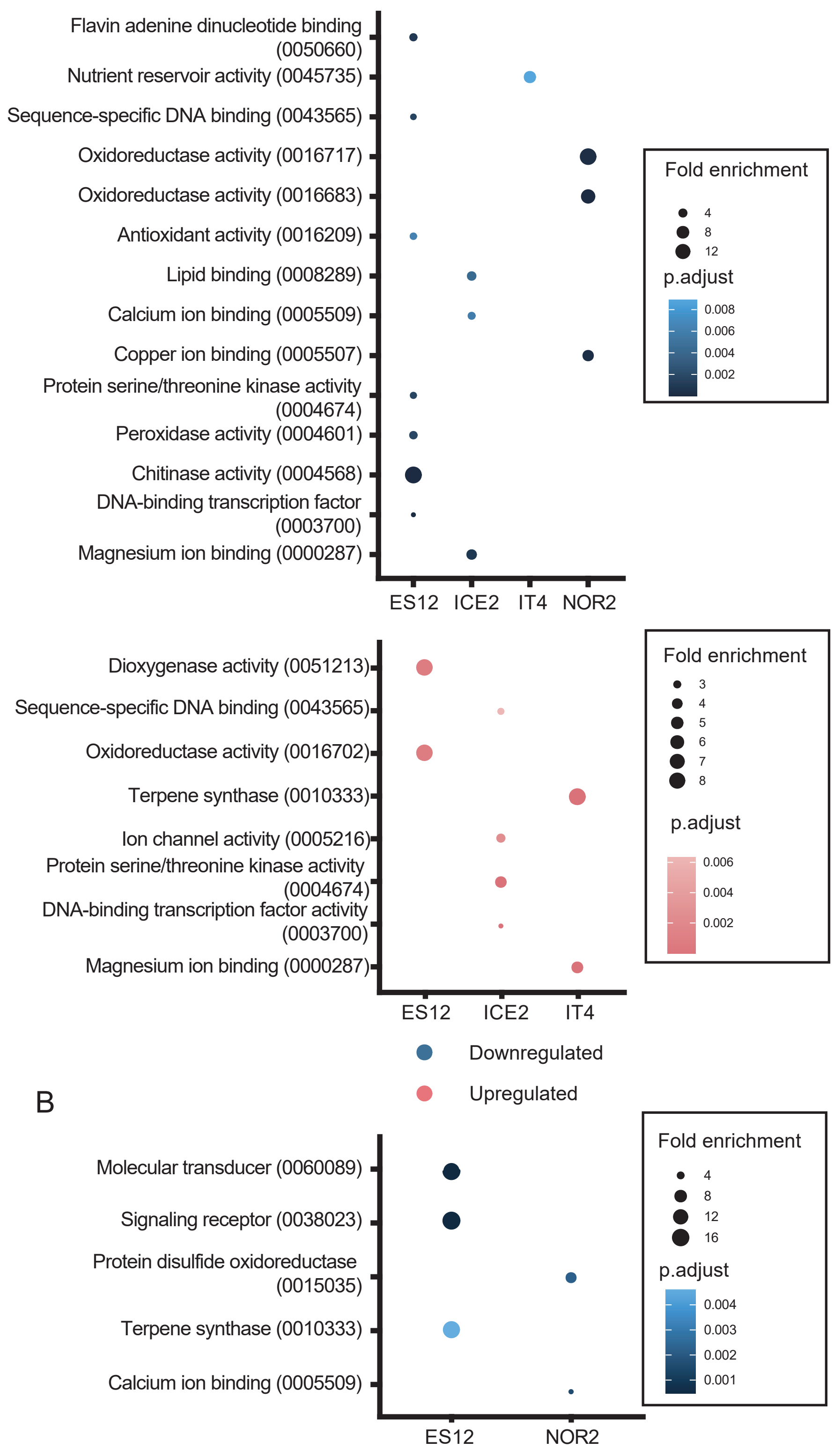

Flavin adenine dinucleotide binding (0050660)
Nutrient reservoir activity (0045735)
Sequence-specific DNA binding (0043565)
Oxidoreductase activity (0016717)
Oxidoreductase activity (0016683)
Antioxidant activity (0016209)
Lipid binding (0008289)
Calcium ion binding (0005509)
Copper ion binding (0005507)
Protein serine/threonine kinase activity (0004674)
Peroxidase activity (0004601)
Chitinase activity (0004568)
DNA-binding transcription factor (0003700)
Magnesium ion binding (0000287)
ES12
ICE2
IT4
NOR2
Fold enrichment
4
8
12
p.adjust
0.008
0.006
0.004
0.002
Dioxygenase activity (0051213)
Sequence-specific DNA binding (0043565)
Oxidoreductase activity (0016702)
Terpene synthase (0010333)
Ion channel activity (0005216)
Protein serine/threonine kinase activity (0004674)
DNA-binding transcription factor activity (0003700)
Magnesium ion binding (0000287)
ES12
ICE2
IT4
Fold enrichment
3
4
5
6
7
8
p.adjust
0.006
0.004
0.002
Downregulated
Upregulated
B
Molecular transducer (0060089)
Signaling receptor (0038023)
Protein disulfide oxidoreductase (0015035)
Terpene synthase (0010333)
Calcium ion binding (0005509)
ES12
NOR2
Fold enrichment
4
8
12
16
p.adjust
0.004
0.003
0.002
0.001

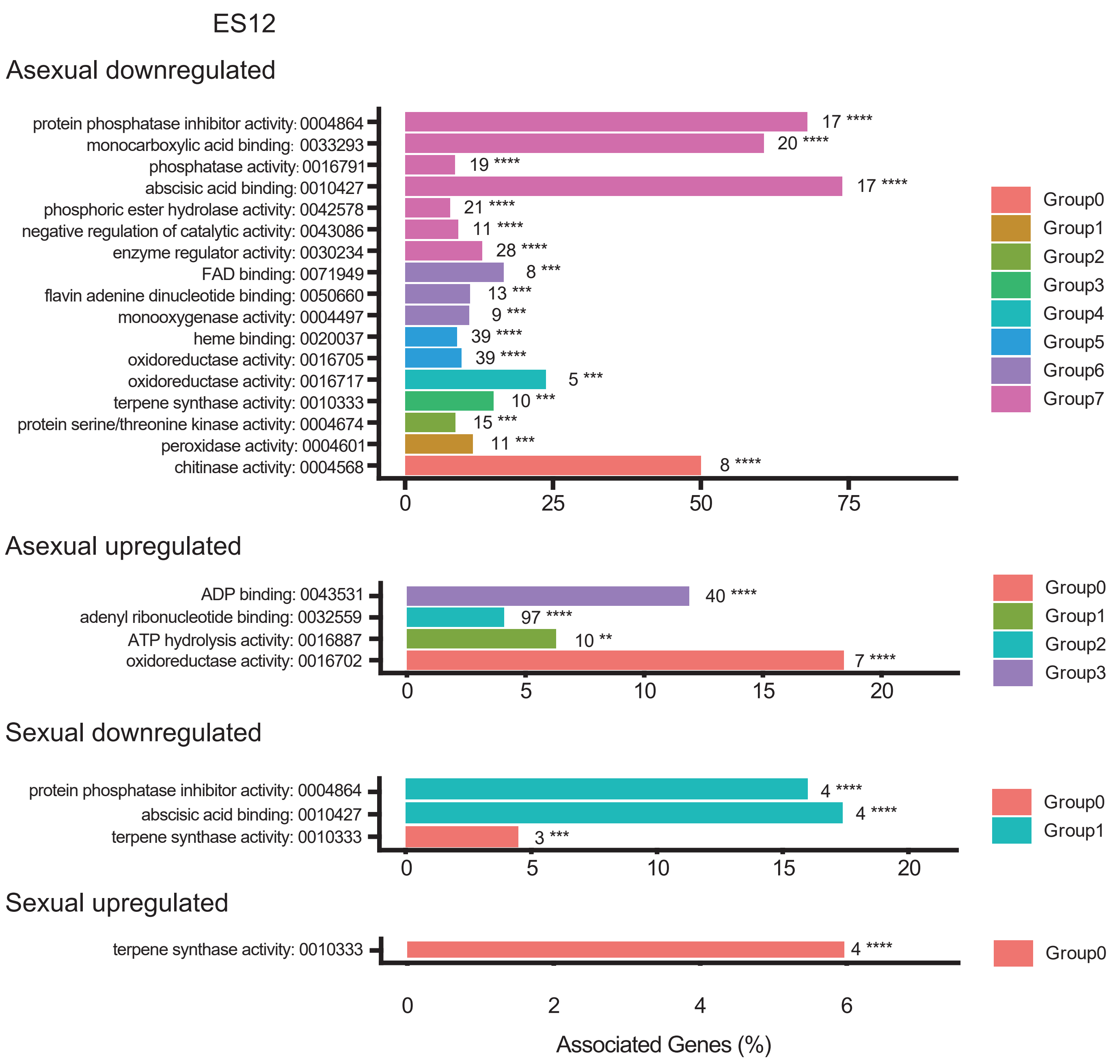
ES12
Asexual downregulated
protein phosphatase inhibitor activity: 0004864
monocarboxylic acid binding: 0033293
phosphatase activity: 0016791
abscisic acid binding: 0010427
phosphoric ester hydrolase activity: 0042578
negative regulation of catalytic activity: 0043086
enzyme regulator activity: 0030234
FAD binding: 0071949
flavin adenine dinucleotide binding: 0050660
monooxygenase activity: 0004497
heme binding: 0020037
oxidoreductase activity: 0016705
oxidoreductase activity: 0016717
terpene synthase activity: 0010333
protein serine/threonine kinase activity: 0004674
peroxidase activity: 0004601
chitinase activity: 0004568
17 ****
20 ****
19 ****
17 ****
21 ****
11 ****
28 ****
8 ***
13 ***
9 ***
39 ****
39 ****
5 ***
10 ***
15 ***
11 ***
8 ****
0
25
50
75
Group0
Group1
Group2
Group3
Group4
Group5
Group6
Group7
Asexual upregulated
ADP binding: 0043531
adenyl ribonucleotide binding: 0032559
ATP hydrolysis activity: 0016887
oxidoreductase activity: 0016702
40 ****
97 ****
10 **
7 ****
0
5
10
15
20
Group0
Group1
Group2
Group3
Sexual downregulated
protein phosphatase inhibitor activity: 0004864
abscisic acid binding: 0010427
terpene synthase activity: 0010333
4 ****
4 ****
3 ***
0
5
10
15
20
Group0
Group1
Sexual upregulated
terpene synthase activity: 0010333
4 ****
0
2
4
6
Group0
Associated Genes (%)

ICE2

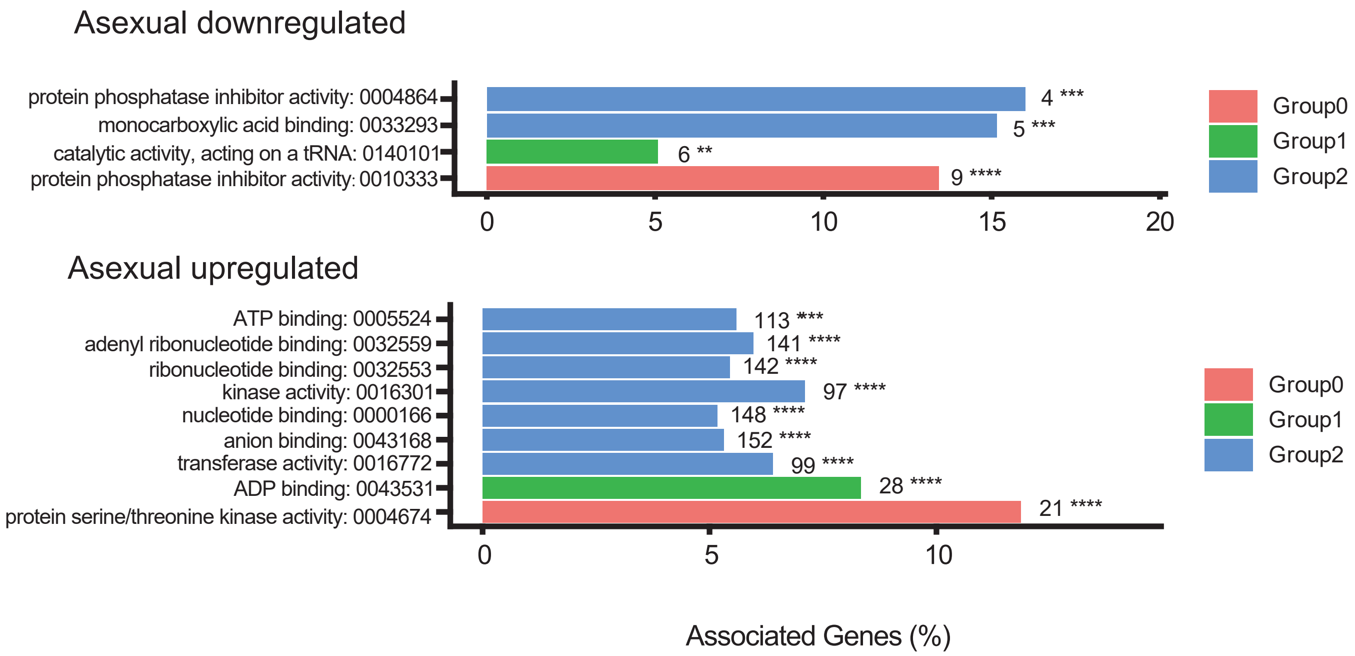
Asexual downregulated
protein phosphatase inhibitor activity: 0004864
monocarboxylic acid binding: 0033293
catalytic activity, acting on a tRNA: 0140101
protein phosphatase inhibitor activity: 0010333
4 ***
5 ***
6 **
9 ****
0
5
10
15
20
Group0
Group1
Group2
Asexual upregulated
ATP binding: 0005524
adenyl ribonucleotide binding: 0032559
ribonucleotide binding: 0032553
kinase activity: 0016301
nucleotide binding: 0000166
anion binding: 0043168
transferase activity: 0016772
ADP binding: 0043531
protein serine/threonine kinase activity: 0004674
113 ***
141 ****
142 ****
97 ****
148 ****
152 ****
99 ****
28 ****
21 ****
0
5
10
Group0
Group1
Group2
Associated Genes (%)

IT4

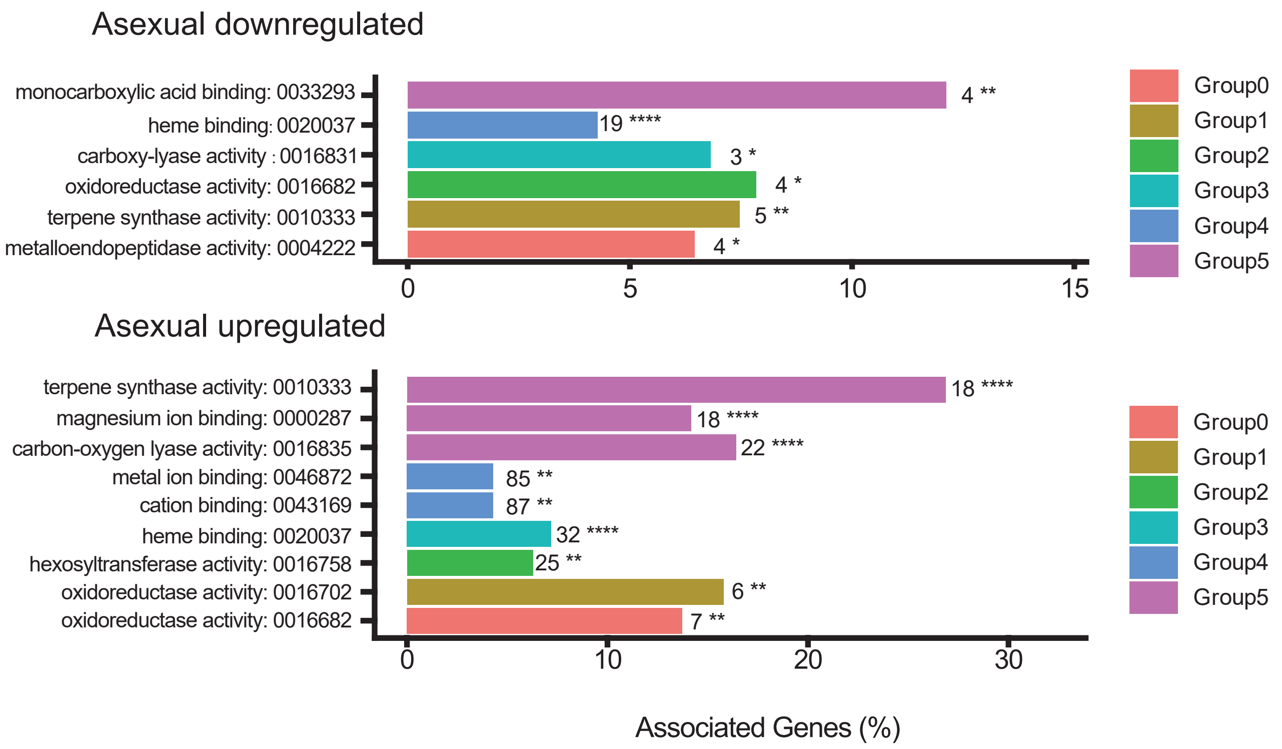

Asexual downregulated
monocarboxylic acid binding: 0033293
heme binding: 0020037
carboxy-lyase activity : 0016831
oxidoreductase activity: 0016682
terpene synthase activity: 0010333
metalloendopeptidase activity: 0004222
4 **
19 ****
3 *
4 *
5 **
4 *
0
5
10
15
Group0
Group1
Group2
Group3
Group4
Group5
Asexual upregulated
terpene synthase activity: 0010333
magnesium ion binding: 0000287
carbon-oxygen lyase activity: 0016835
metal ion binding: 0046872
cation binding: 0043169
heme binding: 0020037
hexosyltransferase activity: 0016758
oxidoreductase activity: 0016702
oxidoreductase activity: 0016682
18 ****
18 ****
22 ****
85 **
87 **
32 ****
25 **
6 **
7 **
0
10
20
30
Group0
Group1
Group2
Group3
Group4
Group5
Associated Genes (%)

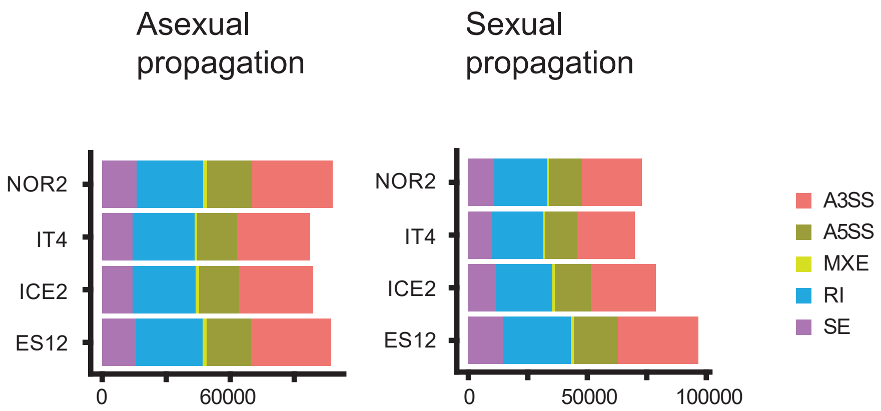

Asexual propagation
Sexual propagation
NOR2
IT4
ICE2
ES12
0
60000
50000
100000
A3SS
A5SS
MXE
RI
SE
